# Forest expansion dominates China’s land carbon sink since 1980

**DOI:** 10.1038/s41467-022-32961-2

**Published:** 2022-09-13

**Authors:** Zhen Yu, Philippe Ciais, Shilong Piao, Richard A. Houghton, Chaoqun Lu, Hanqin Tian, Evgenios Agathokleous, Giri Raj Kattel, Stephen Sitch, Daniel Goll, Xu Yue, Anthony Walker, Pierre Friedlingstein, Atul K. Jain, Shirong Liu, Guoyi Zhou

**Affiliations:** 1 Institute of Ecology and School of Applied Meteorology, Nanjing University of Information Science & Technology, Nanjing, China; 2 Key Laboratory of Forest Ecology and Environment, China’s National Forestry and Grassland Administration, Ecology and Nature Conservation Institute, Chinese Academy of Forestry, Beijing, China; 3 Research Center for Global Changes and Ecosystem Carbon Sequestration & Mitigation, Nanjing University of Information Science & Technology, Nanjing, China; 4 Laboratoire des Sciences du Climat et l’Environnement, CEA CNRS UVSQ Gif-sur-Yvette, Gif-sur-Yvette, France; 5 Sino-French Institute for Earth System Science, College of Urban and Environmental Sciences, Peking University, Beijing, China; 6 Woodwell Climate Research Center, Falmouth, MA USA; 7 Department of Ecology, Evolution, and Organismal Biology, Iowa State University, Ames, IA USA; 8 Schiller Institute for Integrated Science and Society, Department of Earth and Environmental Sciences, Boston College, Chestnut Hill, MA USA; 9 School of Geographical Sciences, Nanjing University of Information Science & Technology, Nanjing, China; 10 Department of Infrastructure Engineering, University of Melbourne, Parkville, Melbourne, Australia; 11 Department of Hydraulic Engineering, Tsinghua University, Beijing, China; 12 College of Life and Environmental Sciences, University of Exeter, Exeter, UK; 13 School of Environmental Science and Engineering, Nanjing University of Information Science & Technology, Nanjing, China; 14 Oak Ridge National Laboratory, Oak Ridge, TN USA; 15 College of Engineering, Mathematics and Physical Sciences, University of Exeter, Exeter, UK; 16 University of Illinois, Urbana-Champaign, Urbana, IL USA

**Keywords:** Carbon cycle, Forestry

## Abstract

Carbon budget accounting relies heavily on Food and Agriculture Organization land-use data reported by governments. Here we develop a new land-use and cover-change database for China, finding that differing historical survey methods biased China’s reported data causing large errors in Food and Agriculture Organization databases. Land ecosystem model simulations driven with the new data reveal a strong carbon sink of 8.9 ± 0.8 Pg carbon from 1980 to 2019 in China, which was not captured in Food and Agriculture Organization data-based estimations due to biased land-use and cover-change signals. The land-use and cover-change in China, characterized by a rapid forest expansion from 1980 to 2019, contributed to nearly 44% of the national terrestrial carbon sink. In contrast, climate changes (22.3%), increasing nitrogen deposition (12.9%), and rising carbon dioxide (8.1%) are less important contributors. This indicates that previous studies have greatly underestimated the impact of land-use and cover-change on the terrestrial carbon balance of China. This study underlines the importance of reliable land-use and cover-change databases in global carbon budget accounting.

## Abstract (web summary)

The impact of land-use and cover-change (LUCC) on ecosystem carbon stock in China is poorly known due to large biases in existing databases. Here the authors develop a new LUCC database with corrected false signals and reveal that forest expansion is the dominant driver of China’s recent carbon sink.

## Introduction

Land-use and cover-change (LUCC) resulting from anthropogenic activities are nearly ubiquitous across Earth’s surface, impacting biogeochemical cycles, and regional and global climate^1^. LUCC-induced carbon (C) emission is one of the most uncertain terms in the global C budget^2,3^, and is thought to be responsible for approximately 25% of the historical atmospheric increase in CO_2_ concentration^4^. To quantify the related emissions, land use change reconstructions are needed, which are used to drive either bookkeeping or Dynamic Global Vegetation Models, e.g. the 5th and 6th Assessment Reports of the Intergovernmental Panel on Climate Change (IPCC) or the annual updates of the Global Carbon Budget^2,5^.

The standard gridded land use change reconstruction used by carbon models is the Land-Use Harmonization (LUH2) dataset^2^. LUH2 is based on Food and Agriculture Organization (FAO) reports of country-level agricultural area disaggregated in space. It integrates the History Database of the Global Environment (HYDE) land use model, with the Miami-LU model to predict the area occupied by forests before human land use^6^, and a simulation of secondary forests area driven by harvest and agricultural land expansion^6^. HYDE represents long-term cropland spatial distribution changes with global coverage, developed by assimilating both inventory and satellite data. Regionally, the distribution of cropland area modeled by HYDE can be biased^7^ however, since it is not fully constrained by observed changes of major land cover types such as grassland and forests. Specifically, the LUH2 dataset was built from national agricultural land area data, but was not constrained by forest area^8^. Sub-national changes of cropland are determined by the HYDE model, using a number of rules and suitability criteria to decide if a pixel is agricultural^9^.

From regional sources, better data can be collected at a fine scale for alternative datasets, such as higher-resolution gridded maps and more accurate measurements. For example, Yu and Lu^10^ and Yu et al.^7^ developed a multi-source harmonized LUCC database for the US and corrected land use change data in LUH2. Such corrections markedly switched LUCC-induced C flux from a strong C sink (−30.3 ± 2.5 Tg C per year, LUH2-based) to a small C source (13.6 ± 3.5 Tg C per year) in the US during 1980–2016.

China has experienced intensive deforestation and afforestation, cropland expansion and abandonment, and grassland and wetland shrinkage and recovery since 1900. Records of land-use history are relatively scarce and sometimes inconsistent^11^. LUCC information has been improved with the use of remote sensing products since the 1980s, but the LUCC’s impact on the C budget of China is uncertain. Earlier assessments of the LUCC impact differed among regions, time periods, and biomes^12–14^. For example, LUCC-induced cumulative C emission in China may differ by a factor of three to five according to different studies (17–33 Pg C vs 6.18 Pg C from 1700 to 2000) based on similar bookkeeping models^12,14^. Such discrepancies mainly stem from the use of different LUCC data^14^. The accuracy and the reliability of the LUCC databases are generally not thoroughly examined, and the spatio-temporal distributions of the major biomes in different databases can be dramatically different. For example, this was the case for the forest areas from LUH2 Global Carbon Budget dataset^15^ (LUH2-GCB hereafter) and the State Forestry Administration of China^16^ (Supplementary Fig. S9). Thus, there is still a lack of long-term comprehensive LUCC databases with known, corrected biases, suggesting that the estimation of LUCC-induced C flux can be further improved. Moreover, model representation of forest C dynamics in China is also generally simplified. None of the earlier studies considered different forest types (i.e., planted or natural forest) and nor was forest management simulated in assessing LUCC-induced C balance. Planted forests (PFs) and natural forests (NFs) should be distinguished because their capacities of C uptake and storage vary considerably due to different species composition, stand structure, age, and impact of management context^17^.

To address these challenges, we have developed a new comprehensive LUCC database addressing known issues by harmonizing multiple sources of inventory data and high-resolution satellite images (see Methods and Supplementary Information 1). Then, we used the database to drive a process-based land ecosystem model (Dynamic Land Ecosystem Model, DLEM) to derive resulting C fluxes. The simulations were compared with MsTMIP and TRENDY, the two most recognized and influential multi-model intercomparison projects. Our goal was to compile an improved data set to assess C storage dynamics in China’s terrestrial ecosystems from 1900 to 2019, with a specific emphasis on the LUCC impacts since 1980 – a period when intensive forest expansion occurred – while also considering contributions of climate, forest management, nitrogen (N) deposition, and CO_2_ fertilization.

## Results and discussion

### Historical land use and cover changes

**Fig. 1:**
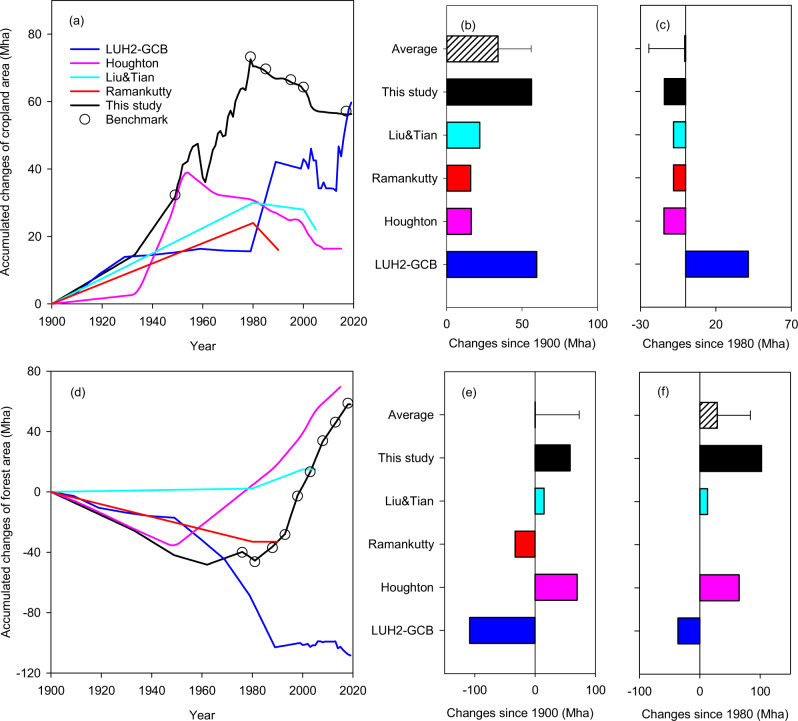
Temporal, net changes of cropland and forest from 1900 (unit: Mha). Panel **a**–**c**: cropland; panel **d**–**f**: forest; the bar charts indicate the total accumulated areas (**b**, **e**) from 1900 and (**c**, **f**) from 1980 until the last available year; LUH2-GCB was the latest version of LUH2 data used in Global Carbon Budget assessments projects (LUH2 used in MsTMIP and TRENDY were showed in Supplementary Figs. S7 and S10); Houghton data were derived from Houghton and Nassikas^20^ and the data in 1900 were interpolated from 1850 and 1950; Liu&Tian and Ramankutty data were derived from the works of Liu and Tian^16^ and Ramankutty and Foley^18^; the open circles indicate the changes of cropland and forest areas derived from inventory-based benchmark data; details of the benchmark data for cropland and forest were presented in Yu et al.^11^ and Supplementary Information 1.2 of this study, respectively; error bars: one standard deviation from the mean.

**Fig. 2:**
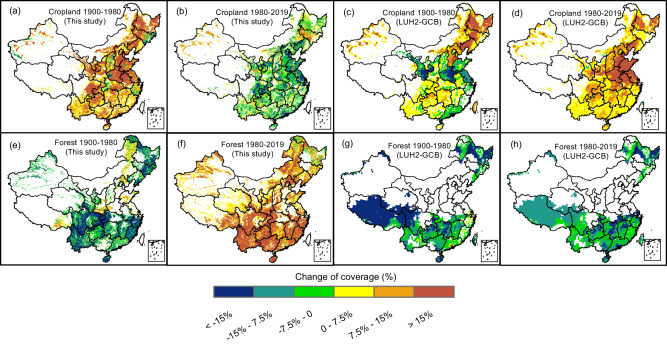
Spatial distribution of the fractional coverage changes of cropland and forest in China (unit: %). Panels **a**–**d**: cropland; panel **e**–**h**: forest; panels **a**, **b**, **e**, and **f** indicate the results derived from this study; data in panels **c**, **d**, **g**, and **h** were from LUH2-GCB; panels **a**, **c**, **e**, and **g** show the changes from 1900 to 1980, whereas panels **b**, **d**, **f**, and **h** show the changes from 1980 to 2019; negative and positive values indicate coverage reduction and increment, respectively.

Existing databases differed significantly in representing historical LUCC in China (Fig. 1). Generally, datasets agree on the direction of change in cropland area until 1980 in Liu and Tian^18^, Ramankutty^19^, Houghton^20^, and this study (Fig. 1b, c), while the magnitude of change varied greatly. Specifically, the total cropland expansion in China was comparable between our new data set and the LUH2-GCB from 1900 onwards (56 vs 60 Mha, Fig. 1b), but cropland area changes since 1980 diverged considerably (−14 vs 41 Mha, Fig. 1c). The differences were also evident across space and more distinct during the period of 1980 to 2019 (Fig. 2a–d), in which the cropland coverage was mainly declining in our reconstructed data but increasing in LUH2-GCB (Fig. 2b, d). We found that the distinct changes are derived from the abrupt cropland increases in the FAO data reported from China, upon which LUH2-GCB was based (see Supplementary Information 3).

The problems of cropland area expansion reported to FAO are likely caused by changes in the underlying database, in which the Chinese Agricultural Yearbook (CAY) was used prior to 1996, the China Land and Resources Statistical Yearbook (LRSY) from 1996 to 2007, and the National Land and Resources Bulletin (NLRB) after 2007 (Supplementary Information 3).

These three datasets are not consistent with each other because surveying methods were distinct. For example, cropland area in CAY before 1982 used an extrapolation method (i.e. “production-to-acreage” approach) due to limited field survey data^11^. Specifically, the extrapolation method was widely adopted for convenience and for taxation purposes in the early period, such as in the framework of the first benchmark cropland survey conducted in 1953. Such methods assumed that low-productivity cropland occupied an area of 1/3–1/8 of a predetermined, “standard-productivity” cropland^21^, which greatly underestimates the acreages of low productivity cropland. Biases accumulated in this method persisted until the satellite era (1980s), while the 1953 surveying data were used as the baseline for CAY to update cropland area on an annual basis.

Besides the survey method, policies also contributed to a bias of reported cropland area. To tackle rising food demands, cropland expansion was highly encouraged by the government before the 1980s, implementing an incentive policy to allow new tax-free cropland without reporting to the government for the first 3–5 years^22,23^. Even after the initial reporting free period, these newly cultivated croplands continued to be unreported due to political incentives to show increasing crop yield to the local authorities^23,24^.

When the first comprehensive and systematic survey (i.e. the second national cropland survey conducted during 1985–1996) was completed, the cropland area was found to be larger than previously reported in CAY^11^. Similarly, the shift from the use of LRSY to NLRB also introduced a spurious cropland area increment from 2007 to 2010 as small, fragmented croplands were identified by better technologies adopted in NLRB, which had remained undetected previously (Supplementary Fig. S10).

Thus, LUH2-GCB has inherited spurious temporal signals of abrupt cropland increment in FAO from the 1980s to 2010 (Fig. 1a and Supplementary Fig. S10). Therefore, if the areas of other land cover types (e.g. forest) are indirectly constrained from cropland area change, cropland area biases were mirrored in the area change of other land use types. This is the case for the LUH2-GCB and for Liu and Tian’s previous land use gridded datasets. Our new database, rebuilt from Yu et al.^11^, corrected these problems in temporal dynamics by assimilating multiple data sources (Fig. 1a). More specifically, we retrospectively reconstructed information about cropland and forest areas year by year, using tabular data from official agencies (Supplementary Information 1 and Supplementary Data 1). To further reduce the aforementioned biases, we used the most recent and authoritative record of provincial cropland and forest areas available as the benchmark, and then spatialized the cropland and forest distributions using gridded maps as ancillary data (Supplementary Information 1). The area changes were also validated using inventory-based benchmark data (Fig. 1a, d, details were presented in Yu et al.^11^ and Supplementary Information 1.2).

Changes in forest area in China also varied dramatically among databases. Based on Ramankutty and Foley^19^ and LUH2-GCB, a net forest loss was found from 1900 to the last available year, at 33–108 Mha whereas Liu and Tian^18^ and Houghton and Nassikas^20^ reported a net increase of 15 Mha (1900–2005) and 70 Mha (1900–2015) in forest area, respectively (Fig. 1d, e).

By assimilating multiple source records, reports, and national surveys, however, our newly reconstructed and intensively validated database (Supplementary Figs. S4, S5, and S8) with corrected biases suggests that the forest area increased by 58 Mha from 1900 to 2019 (Fig. 1e). In particular, our data suggest that there is a surprisingly large underestimation of forest expansion in all other databases (38–102 Mha) after 1980 (Fig. 1f). We performed spatial analyses and show that widespread forest expansion in our reconstructed data was represented as a forest decline in LUH2-GCB during the period 1980–2019 (Fig. 2f, h). These existing biases in the dataset during the last four decades can be simply removed using recently available and spatially explicit forest products (Supplementary Table S2).

Bias in forest change might be explained by two reasons. First, gridded datasets inherited and transferred errors from the use of FAO-based cropland dataset in developing global land use databases such as HYDE and thus LUH2-GCB^8^. Second, the FAO forest area reported is an important reference data used in these databases. The FAO forest area is reported based on a “land use” definition, which underestimated gross “land cover” change signals between reported years (Supplementary Information 1.3). Specifically, the FAO forest area describes lands that have been forested and will continue to be used for forestry (e.g. cut-over area, fired-over area, unestablished afforestation land) (Supplementary Table S5). This approach overestimates forest area by including lands used for reforestation where no forest was yet created. Thus, for example, the FAO statistics reported a 157.2 Mha forest area in 1990 (Supplementary Fig. S7), which is ~30 Mha higher than officially released data.

More importantly, newly established forests were underestimated in such an accounting approach. The forest area expansion in China reported in the FAO statistics was 61 Mha from 1990 to 2019, which is 30 Mha lower than the officially released data^16^. Our reconstructed dataset, in agreement with officially released forest area, uses a “land cover” definition that characterizes the distribution of annually established forests. Therefore, the FAO statistics - a data set with definition specified to describe the area of land use – should be used with caution for constraining the temporal evolution of forest cover distribution in gridded data reconstruction, and the modeling community should be alerted to treat the LUCC data appropriately.

Nonetheless, the FAO and the related LUH2 products were the dominant LUCC forcing data used in multiple studies^3,25^, including various process-model-based intercomparison projects (e.g. MsTMIP, LUMIP, NMIP, TRENDY), annually released Global Carbon Budget reports^2,26^, and IPCC reports^5^, implying a potential bias of these assessments for the China region. In contrast, changes in forest area from our database were independently developed (Supplementary Information 1.2), intensively calibrated, and validated using officially released national forest inventories (NFIs, see Supplementary Figs. S4 and S5), which can help to reduce the potential bias of C balance assessment in China. More specifically, the total forest area and PF area in our database were compared with historical NFIs released by the National Forestry and Grassland Administration at provincial level since 1949 (Supplementary Figs. S4 and S5), which supports the reliability of our reconstructed data.

### Historical carbon stock changes

To illustrate the bias in the C balance of China when using previous LUCC dataset, we performed simulations with the DLEM model for the period 1900–2019 at a resolution of 0.5 × 0.5 degree forced by our new LUCC dataset. We validated the distribution and changes of C stock using published studies and previously reported inventory-based estimations (Supplementary Information 6 and 7). The model could capture well C dynamics in China using inventory-based forest C stock changes at both provincial and national levels as the validation data set (Supplementary Fig. S14).

**Fig. 3:**
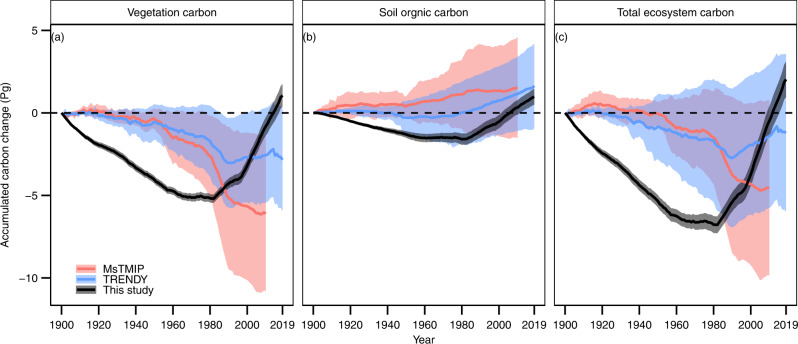
Temporal changes of carbon storage from 1900 to 2010s in China. Panel **a**–**c** indicate vegetation carbon, soil organic carbon, and total ecosystem carbon, respectively. Results derived from experiment designed to have all environmental factors vary historically from 1900 to the 2010s, for model design details of this study see Supplementary Information 8); pink color: MsTMIP (1900–2010); blue color: TRENDY (1900–2019); dark color: this study (1900–2019); the shade areas represent the ranges of 1 standard deviation; unit: Pg C.

Our results show that the total C stock decreased by 6.9 ± 0.6 Pg from 1900 to 1980 and increased by 8.9 ± 0.8 Pg C from 1980 to 2019 (Fig. 3, derived from experiment S1 in Supplementary Table S10). Such a large C stock increment since the 1980s, which is dominated by vegetation biomass C accumulation, was not captured in the MsTMIP and TRENDY projects driven by different versions of the LUH2 data (Fig. 3). This is attributed to the fast expansion of forest area(s) that was not captured by this land use forcing (Fig. 1).

We found that the large-scale forest expansion in China alone has caused a substantial C accumulation since 1980 (0.21 ± 0.006 Pg C per year, Table 1). In contrast, the forest C sink of the TRENDY models is negligible (−0.02 ± 0.05 Pg C per year, Table 1). A moderate C source (0.10 ± 0.08 Pg C per year, Table 1) was even found in the MsTMIP models, since these models were driven by continuous forest area loss and cropland expansion since the 1980s (Supplementary Fig. S7).

**Table 1:** Comparison of reported carbon fluxes from various biomes in China

Category/biome	Method	Reported C flux^a^ (Pg C per year)	Period	Ref.	This study
China	Atmospheric inversion	−1.11 ± 0.38	2010–2016	^27^	−0.28 ± 0.06
China	Process-based model	0.10 ± 0.08 0.04 ± 0.09	1980–2010 2000–2010	MsTMIP	−0.21 ± 0.017 −0.30 ± 0.019
China	Process-based model	−0.02 ± 0.05 −0.03 ± 0.11	1980–2019 2010–2019	TRENDY	−0.23 ± 0.018 −0.28 ± 0.023
China	Atmospheric inversion	−0.26 ± 0.09	2000–2005	^72^	−0.33 ± 0.021
China	Process-based model	−0.12 ± 0.03 −0.26 ± 0.11 −0.29 ± 0.08 −0.18 - −0.24	1981–2000 1996–2005 2000–2005 1961–2005	^13^	−0.16 ± 0.015 −0.29 ± 0.019 −0.33 ± 0.021 −0.10 ± 0.010
China	Inventory estimate + atmospheric inversion	−0.17 ± 0.05	1980–2002	^38^	−0.17 ± 0.016
China	Atmospheric inversion	−0.33	2001–2005	^73^	−0.33 ± 0.020
China	Atmospheric inversion	−0.39 - −0.51 −0.33	2006–2009	^74^	−0.34 ± 0.022
China Forest	Inventory estimate	0.022 −0.021	1949–1980 1977–1998	^29^	0.042 ± 0.001 −0.067 ± 0.010
China Forest^b^	Inventory estimate	−0.055	1973–2003	^75^	−0.063 ± 0.008
China vegetation	Inventory estimate + atmospheric inversion	−0.35 ± 0.33	1996–2005	^38^	−0.22 ± 0.015
China soil^c^	Inventory estimate + atmospheric inversion	−0.075 ± 0.066	1980–2002	^38^	−0.205 ± 0.015
China soil	Process-based model	−0.094 ± 0.047	1981–2000	^13^	−0.055 ± 0.004
Cropland soil	Inventory estimate	−0.026 ± 0.011	1980s,1990s	^76,77^	−0.047 ± 0.003
Grassland soil	Remote sensing + statistical model	−0.007 ± 0.003	1982–1999	^32^	0.062 ± 0.001
Grassland soil	Process-based model	−0.022 ± 0.01	1981–2000	^13^	0.066 ± 0.001

^a^Negative and positive values indicate C sink and source, respectively; ^b^total C sink/source during the period; ^c^includes forest, shrubland, grassland, and cropland only (wetland and urban were excluded). Data reported in this study indicate mean ± 1 standard deviation.

A recent atmospheric inversion-based study reported that China’s land ecosystems were a large CO_2_ sink of −1.11 ± 0.38 Pg C per year^27^, which seems to be ecologically implausible and critically sensitive to the assimilation of the CO_2_ record from one station^28^. The compilation of previous studies from inventory- and satellite-based estimation, atmospheric inversion, and process-based models suggested that the Chinese C sink was much smaller (−0.18– −0.45 Pg C per year; Table 1). Our model-simulated terrestrial sink (~−0.28 ± 0.06 Pg C per year) was in this range (Table 1).

While our simulated C balance in different categories or biomes is close to previous estimations, three major differences are observed (Table 1). First, because the LUCC data used in previous global models suffered from biases as shown above, the national C sink was generally underestimated in these simulations (Table 1). Second, our estimation of the forest sink is around two to three times larger than the previous one during 1949–1998^29^. This was mainly because forest area was underestimated by over 33% (53 Mha) in the previous study^29^ compared to the national forest inventory (NFI)^16^. This underestimation may stem from exclusion of economic and bamboo forests. The third major difference is the role of grassland soils in C balance during the period 1980–2000. China’s grassland soils were previously reported as a minor sink of −0.007–−0.022 Pg C per year from the 1980s to the 2000s (Table 1), while our simulations suggest that grassland soils were a C source of 0.062–0.066 Pg C per year. This discrepancy lies in the approaches used and the accounting boundaries between studies (i.e. whether the transitions of grassland were considered), in which LUCC impacts were represented differently. For example, impervious surfaces (part of urbanized area) expanded into ~15 Mha of natural lands in China from 1978 to 2017^30^, which further drove redistribution of cropland into marginal lands with the majority converted from grassland, causing wind erosion, habitat loss, and more water and fertilizer consumption^31^. Earlier studies using a static grassland map exclude the C stock loss in the land-use transition^32^. Thus, the distinct roles of grassland soils (i.e. sink vs source) derived from our simulations and earlier studies are not contradictory but are due to differences in accounting boundaries.

### LUCC impacts on carbon stock changes

**Fig. 4:**
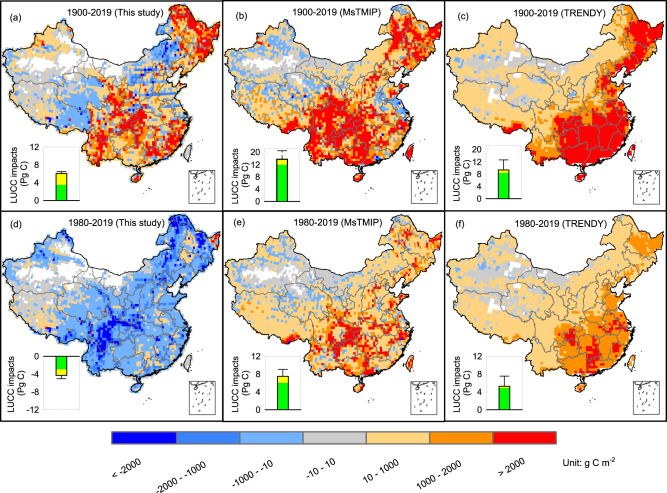
Spatial distribution of LUCC impacts on ecosystem carbon storage. Panel **a**–**c**: LUCC impacts for period of 1900–2019; panel **d**–**f**: LUCC impacts for period of 1980–2019 (**d**–**f**). Panels **a** and **d** are from this study; data in panels **b** and **e** are from MsTMIP; data in panels **c** and **f** are from TRENDY; negative and positive values indicate sink and source, respectively; green and yellow bar stacked in the insert indicate LUCC impacts on vegetation and soil organic carbon in Pg C; spatial map unit: g C m^−2^; error bars: one standard deviation from the mean of LUCC impacts on total carbon storage.

Our DLEM simulation indicates that LUCC induced a C loss of 5.1 ± 0.7 Pg C from 1900 to 2010s (Fig. 4a), which is substantially lower than that from MsTMIP (13.8 ± 7.7 Pg C, 1900–2010) and TRENDY (9.4 ± 3.3 Pg C, 1900–2019; Fig. 4e, f and Supplementary Fig. S18d, g). From 1980 onward, LUCC increased C storage by 4.3 ± 0.7 Pg C, with the major contribution from vegetation biomass C increment in the southwestern and northeastern regions (Fig. 4d and Supplementary Fig. S19a). Nonetheless, this C increase in biomass was not captured in MsTMIP and TRENDY models (Fig. 4e, f and Supplementary Fig. S19d, g), which simulated that LUCC continued to reduce C stock by 7.5 ± 1.6 and 5.3 ± 2.3 Pg C during the period 1980 to the 2010s, respectively (Fig. 4 and Supplementary Fig. S20).

To confirm that such discrepancy was induced by LUCC data but not the DLEM model, we set up additional DLEM simulations using the LUH2-GCB database (Supplementary Information 8). The simulated C losses induced by LUCC when DLEM was driven with LUH2-GCB were 6.5 ± 0.4 and 11.4 ± 0.6 Pg C during the periods of 1980–2019 and 1900–2019, which are close to MsTMIP and TRENDY simulations. These results confirm that the LUCC forcing database is the major contributor to the difference between our simulations and the MsTMIP and TRENDY projects. An earlier study reported that global LUCC-induced C emissions are substantially underestimated due to underrepresented tree harvesting and land clearing from shifting cultivation^33^. Our simulation revealed that regional LUCC-induced C emission could also be overestimated in China due to a bias in the LUCC data.

There are also disputes over whether the LUCC induced a C sink in China since the 1990s or not (Supplementary Table S8). By using an updated LUCC database, our simulations revealed that LUCC was a strong C sink in China, and that its magnitude was larger than previous estimates since the 1990s (Supplementary Table S8). Our results using an improved LUCC forcing data can facilitate narrowing down the well-known, large uncertainty in LUCC-induced C change at regional scale.

### Attributions of different factors on C stock changes since 1980

**Fig. 5:**
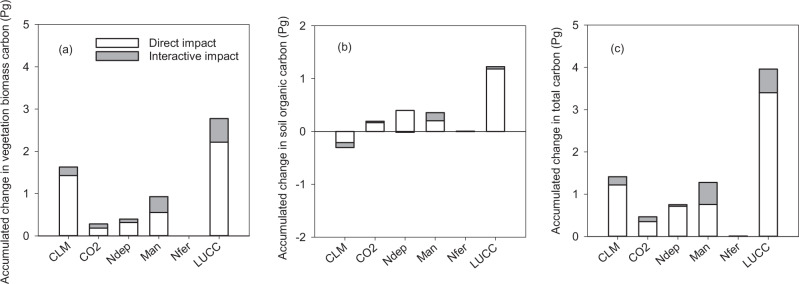
Attributions of different environmental factors on carbon stock change in China from 1980 to 2019. Panels **a**–**c** indicate attributions of impacts on the changes of vegetation carbon, soil organic carbon, and total ecosystem carbon, respectively; CLM: climate; CO2: rising atmospheric CO_2_ concentration; Ndep: N deposition; Man: forest management; Nfer: N fertilizer and manure application.

By using the DLEM model with factorial simulations (see Supplementary Information 8 for details), we examined the direct and interactive contributions of different drivers to terrestrial C stock change in China for the period 1980–2019, including LUCC, climate, forest management, N deposition, and CO_2_ fertilization (see Methods, Fig. 5). Note that historical C stock change is not equivalent to the sum of factorial attributions as the baseline conditions differ (see Supplementary Information 8).

Overall, 81.9% (6.5 Pg C) of the terrestrial C sink during this period was attributed to direct impacts of all major factors, while the interactive effect contributed 18.1% (1.43 Pg C; Fig. 5c). Among all the factors examined, LUCC was the dominant driver accounting for 50.3% (3.96 Pg C) of the total C increment during the period 1980–2019 (Fig. 5c), which was largely attributed to biomass C accumulation (70.0%; Fig. 5a, c). Tian et al.^13^ reported that LUCC’s contribution to the sink in China was at 0.05 Pg C yr^−1^ since the 1980s – an amount that is only about 30% of our simulations. The discrepancy is attributed to the different representation of forest expansion in model simulations, which was 65 Mha from 1980 to 2005 in our database but only ~14 Mha in Tian et al.^13^. Similarly, the increase in the global land sink during the recent period (1998–2012) was also mainly attributed to LUCC (i.e. decreased tropical forest area loss and increased afforestation in northern temperate regions), instead of CO_2_ or climate change^34^.

Climate change enhanced biomass C stocks by 1.63 Pg but caused a soil C loss of 0.30 Pg, thus contributing to land sink of 1.41 Pg C (18.0% of the total with all factors) since 1980 (Fig. 5). Other global change factors, such as N fertilizer application, atmospheric N deposition, and rising CO_2_, had a relatively minor contribution (0.1–9.54%) to the terrestrial C sink. Therefore, conversely to previous studies^13,35–37^, we showed that LUCC was the dominant driver of the recent land C sink in China, and other factors including climate change, rising CO_2_, and N deposition, contributed much less (0.1–18.0%) to the C stock increment in China (Fig. 5c). Tian et al.^13^ pointed out that LUCC effects in China should not be ignored and that the CO_2_ fertilization effect might be overestimated in Piao et al.^38^.

Our simulations confirm these statements, and further show that LUCC was actually the largest contributor to land sink in China since 1980 (Fig. 5). In those studies which did not account for the influence of LUCC separately, the effects of other global change factors may have been overestimated by including LUCC impacts. For example, Chen et al.^39^ and He et al.^37^ attributed China’s C sink into different components including climate change, leaf area index (LAI) change, rising CO_2_, and N deposition. Such partition inevitably masked the separate contribution from LUCC, because LAI changes are closely related to land-cover changes. Thus, the accurate representation of the LUCC should be prioritized in future modeling attribution studies.

### Carbon stock changes in each land cover type since 1980

**Fig. 6:**
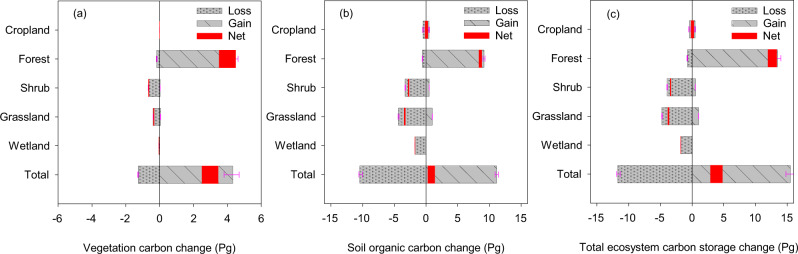
LUCC-induced carbon storage changes by land cover types based on model simulations during 1980–2019. Panel **a**–**c** indicate vegetation carbon, soil organic carbon, and total ecosystem carbon, respectively; the widths of the red blocks indicate the estimation ranges of net changes in model simulations; purple error bars indicate one standard deviation of multiple model runs; negative and positive changes indicate carbon loss and gain, respectively.

The contribution of the establishment of young and new forest plantations to C sink has received increasing attention^3,40–42^. Our simulation (experiment S1, see Methods section) revealed that the increase in terrestrial C stock was dominantly contributed by biomass C accumulation (76.3%) (Fig. 5), in which the natural and planted forests accounted for 65% (2.9 Pg C) and 35% (1.6 Pg C) during the last four decades. We examined the LUCC effect (i.e. the largest contributor of C stock increment in Fig. 5) on the C stock of different biomes and confirmed that forest was the major contributor of the net C accumulation in China since 1980, while other biomes, including cropland, grassland, shrubland, and wetland, were relatively stable, varying from −0.3 to 0.3 Pg C during the same period (Fig. 6). A recent study documented that forest expansion was essential for a large C sink in southern China during 2002–2017, where newly-established and existing forests contributed to 32% and 34% of land C sink in the region^43^. In comparison to the large biomass C increase since 1980 (3.0 Pg C, Fig. 6a), the SOC increase was much lower (0.7 Pg C) during the concurrent period, although SOC changes in each biome varied greatly (–3.4–8.6 Pg C; Fig. 6b) due to area change from land conversions. The biome-level analyses further revealed that the LUCC-induced C stock increment was dominantly contributed from forest and by area expansion, while C storage in grassland and shrubland was reduced by LUCC (Fig. 6).

This study highlights the dominant role of LUCC in determining the terrestrial C sink in China. Because of inaccurate representations of land cover change in China, previous estimates of the terrestrial C sink have been strongly underestimated. In contrast, forest expansion and cropland abandonment have been overestimated in the U.S., resulting in an underestimated C emission since 1980^7^. Hence, we highlighted that the global LUCC database should be further improved, which could potentially narrow down the C imbalance reported in global C budget accounting^2^. In contrast to the previous studies, we showed that the contributions of factors including rising CO_2_, N deposition, and climate change to the land C sink in China were much smaller than LUCC over the past four decades (1980-present time). Thus, reforestation projects could represent important climate change mitigation pathways, with co-benefits for biodiversity^33^. To achieve the ‘C neutrality’ goal as the Chinese government declared, future climate policy should be directed to improve land management, especially forest ecosystems.

### Implications for future LUCC data improvements

This study provides a novel reconstruction of recent land use change in China and assesses its implications in quantifying for terrestrial C storage dynamics. The improved dataset more accurately depicts the spatiotemporal dynamics of LUCC in China because the historically contradictory surveying records were identified, which helped to correct the biased temporal signals. Specifically, the improved surveying methods and the socioeconomic factors have greatly shaped the LUCC signals. We advocate that these impacts should be considered in the reconstruction of the national and global LUCC dataset, especially in the areas that have been intensively disturbed by human activities as is the case of China. These endeavours will be worthwhile, as demonstrated by the large impact that these bias corrections have on China’s C dynamic assessments since 1900. Thus, accurate delineation of LUCC forcing should be stressed in global simulations, including C budget accounting, biodiversity assessments, and ecosystem services evaluations.

## Methods

To quantify and attribute factors affecting C stock changes in China, we examined simulation results from MsTMIP and TRENDY. Both MsTMIP and TRENDY projects provide factorial experiments designed to quantify the impacts of each major environmental driver on C stock changes, such as climate, land use, atmospheric CO_2_, and N deposition^44–46^.

For comparison and quantification of the impacts of LUCC on C stock using different databases, we used DLEM - a process-based biogeochemical model used in both model intercomparison projects of MsTMIP and TRENDY (v9) – to examine the impacts of land-use forcing data on the C budget in China. The DLEM model has been widely acknowledged for its regional and global C storage estimations^7,47–50^, and has contributed to the 2013 US national climate assessment report, the MsTMIP, the global N_2_O Model Intercomparison Project (NMIP), the TRENDY project (v9), and the Global Carbon Budget assessments^2^. The model is driven by atmospheric chemistry (i.e. CO_2_, N deposition), climate, forest management, and land-use change at a daily time-step from 1900 to 2019 at a resolution of 0.5 × 0.5°.

### Newly-developed land-use and cover-change datasets

The historical, gridded land-use datasets were developed using multiple sources of data, including gridded images from 1887 to 2019, vector maps in 1980s, and tabular data from 1949 to 2018 (Supplementary Table S1). Specifically, the distribution of lake, river, and barren areas was derived from the GlobeLand30 produced by the Ministry of Natural Resources of China (https://lcviewer.vito.be/download), and we assumed that these land cover types remained unchanged since 1900 (Supplementary Table S2). The impervious land was directly resampled from published data covering the period 1978–2017^30^, while the periods 1900–1977 and 2018–2019 were assumed to be the same as in 1978 and 2017, respectively (Supplementary Table S2). Other vegetation cover types, including cropland, forest, wetland, grassland, and shrubland were reconstructed individually. To do so, we developed a top-down model to reconstruct the historical distribution of cropland, forest, and wetland in China spanning the period 1900–2019. The model was upgraded from the previous version^10^ by allocating a specific, prior-determined, provincial-level area of different biomes to grids in China. The newly reconstructed LUCC database assimilates land conversion signals from reports, field surveys, and satellite images (Supplementary Table S1 and Supplementary Data 1).

The newly reconstructed LUCC database was validated with both provincial statistics and remote-sensing product in a spatial framework. Specifically, forest and cropland areas were validated at each province using historical NFIs, annual yearbooks from the National Forestry and Grassland Data Center (NFGDC), and publications (forest validation: Supplementary Figs. S2–S5, cropland validation: Yu et al.^11^). Besides, the forest and cropland maps were compared with other gridded products (e.g. HYDE, LUH2-GCB) in the years of 1980, 1990, and 2018 (Supplementary Figs. S8 and S9). Our reconstructed maps are more consistent in depicting forest and cropland distribution if the remote-sensing images are used as a reference. More details can be found in Supplementary Information 1.

### Forest distribution, type, age, and harvesting datasets developed

The structure, composition, and dynamic of natural and planted forests are distinct. In this study, we separated NF and PF from the previously developed forest distribution dataset, which facilitated the representation and implementation of forest management in simulations. Similar to the construction of LUCC datasets, the annual provincial PF and NF areas were determined before developing the spatial distribution maps. The provincial-level forest areas were obtained from the NFI data officially released by the State Forestry Administration of China from 1949 to 2018 (see Supplementary Table S3), while the data for the period 1900–1948 were linearly interpolated from Yang et al.^51^ The PF areas were obtained from the NFI and Liu et al.^52^ study covering the period 1973–2018; however, the period 1900–1972 was extrapolated using historical records (see Supplementary Information 1.2). The annual NF time-series were the differences of total forests and the PF. Then, we used the same model developed previously^53^ to reconstruct PF distribution in each base year from 1900 to 2019 (see Supplementary Information 1.2), while the years between the base years were linearly interpolated. Historical distributions of NF, PF, and all forests are presented in Supplementary Figs. S2 and S3. Besides, forest age and type maps of NF and PF were obtained from our previous study^53^.

Forest harvesting information were obtained from the LUH2 land transition dataset (https://daac.ornl.gov/VEGETATION/guides/LUH2_GCB2019.html). The annual, spatial-explicit harvesting data cover the entire study period (1900–2019) at a 0.5° resolution. We then split the annually harvested forest area into NF and PF by the harvesting ratio obtained from annual Yearbooks and publication^54^. The harvesting ratio of the entire study period can be divided into 13 periods (see Supplementary Information 5). Since data were not available before 1949, we assumed that NF and PF harvesting intensities (ratio) were the same as the earliest period available (i.e. 1950–1962). For the period after 2004 when no data were available either, we assumed that NF harvesting gradually decreased while PF harvesting increased since China has policies enforced to shift forest harvesting to PF^55^. The protocol implemented in C loss from forest harvesting is explained in Supplementary Information 5, and the model simulated C loss from forest harvesting was showed in Supplementary Fig. S13.

### Crop rotation and fertilizer datasets

Crop rotation maps were obtained from Liu et al.^56^ for the period 1980–2011, while the crop type during the periods before 1980 and after 2011 was set constant to the nearest year available. Historical, crop specific N fertilizer use rates were obtained from the FAO website (http://www.fao.org/faostat/) and the study of Li et al.^57^ Annual manure applications in cropland were obtained from the study of Zhang et al.^58^

### Climatic and atmospheric chemical condition datasets

We reconstructed daily climate data from meteorological stations and published datasets available for the period 1900 to 2019. Specifically, the maximum, minimum, and average air temperature as well as the precipitation data were derived from observations from 839 meteorological stations and the historical monthly gridded data of Peng et al.^59^ For the period 1980–2019, the daily climatic factors were spatially interpolated using the Anusplin software (Ver. 4.1; Australian National University, Center for Resources and Environmental Studies, Canberra, Australia), according to the approach elaborated in Yu et al.^60^ For the period 1900–1979, we derived the daily climatic factors using the monthly gridded data of Peng et al.^59^ and the daily change pattern of the interpolated images from the meteorological station in 1980.

Similarly, the reconstruction of the shortwave radiation data was also divided into two periods. For the first period from 1984 to 2019, the radiation dataset was downloaded and resampled from the global surface solar radiation dataset at 3-h, 10-km resolution provided by the National Tibetan Plateau Data Center^61^. For the second period from 1901 to 1983, radiation data were obtained from the high-resolution gridded data products provided by the North American Carbon Program Multiscale Synthesis and Terrestrial Model Intercomparison Project (NACP MsTMIP)^62,63^.

Other atmospheric chemical components, including atmospheric CO_2_ concentration, and nitrogen deposition data, were retrieved from IPCC historical CO_2_ data and the NACP MsTMIP (https://daac.ornl.gov/NACP). We also updated N deposition maps for the period of 1996 to 2015 using the product provided by Jia et al.^64^, which was also served as the baseline to proportionally adjust the period before 1996. All the datasets were prepared at or resampled to 0.5 × 0.5° for simulations.

### Model validation

The DLEM model has been intensively calibrated and validated at various temporal scales ranging from a few days to hundreds of years^7,50,65–67^, and spatial levels from sites to globe^47,50,68–70^. This study also conducted rigorous model calibration of biomass and soil C stock using measurement data collected from a nationwide field campaign in China. The field campaign was conducted in 2011–2015, during which the information of geographical characteristics, forest origins (natural or planted forests), soil properties, vegetation properties, disturbances, and PF management were recorded^17,53^ (see Supplementary Information 4). The model validation results can be found in Supplementary Fig. S11.

### Experimental design and statistical analysis

In this study, we set up simulations to distinguish and quantify the effects of LUCC, climate, CO_2_, N deposition, and forest management on terrestrial C storage change in China from 1900 or 1980 to 2019 (see Supplementary Fig. S21 for approached used to quantify LUCC impacts). We first obtained the initial condition of each biome in each grid cell (equilibrium state), which is defined as the interannual variations of a 20-year net flux of C, N, and water less than 1 g C m^−2^ per year, 1 g N m^−2^ per year, and 1 mm m^−2^ per year, respectively^7,50^. A 10-year spin-up run was applied before the transient run using initial state information obtained from the equilibrium run, which helps avoiding abrupt changes resulting from mode transition. The transient run was forced by various drivers designed specifically (Supplementary Table S10).

We designed three groups of experiments to quantify each major driver’s impacts on the terrestrial C stock. Specifically, the first group includes two experiments (Group-1 in Supplementary Table S10), which were used to examine the historical accumulated impacts of LUCC on the terrestrial C stock over the entire study period from 1900 to 2019. Two additional groups of experiments (Group-2 and 3 in Supplementary Table S10) were also designed to quantify the effects of each major driver for the recent four decades (1980–2019), during which the forest expansion was initiated by the Chinese government. Specifically, the two groups of experiment simulations were designed to examine the direct and interactive contributions of each major driver (e.g. LUCC, climate, N deposition, rising CO_2_, and forest management) to the changes of terrestrial C stock in China since 1980. For example, Group-2 experiments (S3-S8 in Supplementary Table S10) were designed to keep a specific environmental factor fixed at the 1980 level, while varying other drivers during the entire study period. Conversely, Group-3 experiments (S9-S15 in Supplementary Table S10) were designed to let at most one environmental factor vary during the period 1980–2019, while keeping the other factors constant at the 1980 level. By keeping a particular environmental factor constant at the 1980 level in Group-2 experiments, the direct impact of the factor and the interactive effects of the fixed factor with other factors were excluded. Therefore, by comparing Group-2 experiments and S1, we are able to quantify the total effects of the particular driver, including both direct and interactive effects. On the other hand, by allowing a specific environmental factor to vary while keeping all other factors fixed at the 1980 level, the interactive effects of the factor were excluded. Thus, the direct effects attributed solely to the specific factor can be quantified by comparing S10 and Group-3 experiments, and the interactive impacts can be derived from Group-2 and Group-3 experiments. Additionally, for uncertainty analysis, we designed two more sets of experiments (Supplementary Table S10) with parameters varying by 1 standard deviation. All the simulations were performed at a 0.5 × 0.5°. Due to limited available experiments designed in MsTMIP and TRENDY, the LUCC-induced C changes are different as their simulations have different interactive effects included. For example, in MsTMIP, the LUCC impact on C storage changes were derived from simulation ‘SG1’ (time-varying climate and constant CO_2_ and N deposition combined with no LUCC) and ‘SG2’ (time-varying climate and constant CO_2_ and N deposition in combination with historical LUCC) (https://nacp.ornl.gov/). While in TRENDY, LUCC impacts were derived from simulation ‘S2’ (time-varying climate and CO_2_ in combination with no LUCC) and ‘S3’ (time-varying climate and CO_2_ combined with LUCC) (https://sites.exeter.ac.uk/trendy).

Note that historical C sink/source of a period was derived from the initial and final years in experiment S1, while the attributions were derived from the factorial simulations (e.g. S3-S8, S10-S15) and a baseline simulation (e.g. S1 for S3-S8, S9 for S10-S15; Supplementary Fig. S21). Similar to former studies^7,50^, we quantified uncertainty sources from model parameters (i.e. LUCC-induced instantaneous C emission) and cropland management (i.e. crop residue return). We also considered uncertainties introduced by NF and PF management. Various forest management practices have been applied in China’s forest. Specifically, NFs may be improved by tending, conservation (close hills to facilitate natural growth), and recovery of degraded forests. For PFs, management practices such as thinning, shrub/grass removal, tending, fertilization, and irrigation may be implemented^53,71^. The management type, intensity, and distribution, however, were unknown for both NFs and PFs. We hereby designed two types of simulations with forest management incorporated or not in NFs and PFs. More specifically, for simulations considering that the forest is managed, we assumed that management helps to increase N uptake ability by 20% in the NFs. For PF, we assumed that (1) irrigation was implemented; (2) fertilizer was applied annually during the first 5 years after planting and 3 years before reaching mature stage at a rate of 15 g N m^−2^ per year; and (3) management helps to increase N uptake ability by 20% in the PF due to understory harvesting and tending.

## Supplementary information

Supplementary Information

Description of Additional Supplementary Files

Supplementary Data 1

## Data Availability

The reconstructed LUCC data used in this study are provided along with this paper. The TRENDY datasets can be requested from S. Sitch (s.a.sitch@exeter.ac.uk) and P. Friedlingstein (p.friedlingstein@exeter.ac.uk). The MsTMIP data are available from the Oak Ridge National Laboratory Distributed Active Archive Center (10.3334/ORNLDAAC/1225).

## Code availability

The code used in this study is available from the corresponding author on request.
