# Supplementary material for: Forest expansion dominates China’s land carbon sink since 1980
Source: Nat Commun. 2022 Sep 13;13:5374. doi: 10.1038/s41467-022-32961-2 (PMC9470586; doi:10.1038/s41467-022-32961-2)
Supplement: Supplementary file 1 — Supplementary Information [file 41467_2022_32961_MOESM1_ESM.pdf]

Supplementary Information for

Forest expansion dominates China's land sink since 1980

Zhen Yu[a,b,c], Philippe Ciais[d], Shilong Piao[e], Richard A. Houghton[f], Chaoqun Lu[g], Hanqin Tian[h], Evgenios Agathokleous[a], Giri Raj Kattel[i,j,k], Stephen Sitch[l], Daniel Goll[d], Xu Yue[m], Anthony Walker[n], Pierre Friedlingstein[o], Atul K. Jain[p], Shirong Liu[b]*, Guoyi Zhou[a]*

[a] *Institute of Ecology and School of Applied Meteorology, Nanjing University of Information Science & Technology, Nanjing, China*

[b] *Key Laboratory of Forest Ecology and Environment, China's National Forestry and Grassland Administration, Ecology and Nature Conservation Institute, Chinese Academy of Forestry, Beijing, China*

[c] *Research Center for Global Changes and Ecosystem Carbon Sequestration & Mitigation, Nanjing University of Information Science & Technology, Nanjing, China*

[d] *Laboratoire des Sciences du Climat et l'Environnement; CEA CNRS UVSQ Gif-sur-Yvette, France*

[e] *Sino-French Institute for Earth System Science, College of Urban and Environmental Sciences, Peking University, Beijing, China*

[f] *Woodwell Climate Research Center, Falmouth, Massachusetts, USA*

[g] *Department of Ecology, Evolution, and Organismal Biology, Iowa State University, Ames, IA, USA*

[h] *International Center for Climate and Global Change Research and School of Forestry and Wildlife Sciences, Auburn University, Auburn, Alabama, USA*

[i] *School of Geographical Sciences, Nanjing University of Information Science & Technology, Nanjing, China*

[j] *Department of Infrastructure Engineering, University of Melbourne, Parkville, Melbourne, Australia*
[k] *Department of Hydraulic Engineering, Tsinghua University, Beijing, China*
[l] *College of Life and Environmental Sciences, University of Exeter, Exeter, UK*
[m] *School of Environmental Science and Engineering, Nanjing University of Information Science & Technology, Nanjing, China*
[n] *Oak Ridge National Laboratory, Oak Ridge, TN, USA*
[o] *College of Engineering, Mathematics and Physical Sciences, University of Exeter, Exeter, UK*
[p] *University of Illinois, Urbana-Champaign, Urbana, IL, USA*
***Email:** liusr@caf.ac.cn; gyzhou@nuist.edu.cn

This supplementary file includes:

1) Supplementary information 1: Reconstruction of LUCC forcing data in China
2) Supplementary information 2: Comparison of LUCC data used in different studies
3) Supplementary information 3: Biases in FAO and LUH2 cropland datasets
4) Supplementary information 4: Field measured sites used
5) Supplementary information 5: Forest carbon loss from harvesting
6) Supplementary information 6: Validations of the changes in simulated forest carbon stock
7) Supplementary information 7: Distribution of simulated biomass C
8) Supplementary information 8: Experiments designed for model simulations
9) References

1. Reconstruction of LUCC forcing data in China

1.1 Datasets used for reconstruction of LUCC forcing data in China

Our reconstructed database depicts historical land cover changes in China. We used a previously developed model to reconstruct the spatial distribution of each major land-cover type in China individually. Major land-cover types were classified as non-vegetation (e.g. impervious land, water, bareland, and snow and ice land) and vegetation (e.g. cropland, forest, grassland, wetland, and shrubland). Each land-cover type was represented by a percentage of area in gridcells and varied annually. More specifically, in the reconstruction of the land-cover dataset of a vegetation type (a biome such as forest), we first reconstructed the annual provincial area of the biome from reports, surveys, publications, and satellite images (Table S1). Second, we built weighting maps representing the potential (possibility) of the biome by assimilating various gridded data for different time windows in the period 1900-2019. Third, the area of the biome was allocated spatially according to the weighting map to match the provincial records. The datasets used in the reconstruction of each land-cover type are listed in Table S2. All the data were developed with a 1-km resolution and resampled to 0.5 $\times$ 0.5 degree for model simulations.

Using cropland as an example, we divided the reconstruction into six periods according to the data availability [1]. In the first step, we adopted the most recent (in 2017) and authoritative records of provincial cropland acreage officially released by the Ministry of Land and Resources of China as the benchmark data. The annual amount of national cropland areas during historical years were reconstructed by adjusting the benchmark year data using inter-annual cropland change information derived from different sources. In the second step, we built a potential cropland map (weighting map) using the gridded images available during each of the periods, including remote sensing products, weighting image of the distance to the nearest urban and rural land, and the cropland constructed in each of the previous years. In the last step, the cropland abandonment and expansion were triggered in each year according to the annual changes of cropland area reconstructed, while the locations were determined using the weighting map. To sum up, this reconstructing model is developed based on the idea of allocating a

prescribed cropland area to each province with priority given to gird cells that have a higher possibility. More details can be found in previous studies [1,2], while the reconstructed cropland dataset has been made available to the public via an open-data repository (https://doi.org/10.6084/m9.figshare.13356680) [1].

Table S1. Datasets used for land cover data reconstruction in China

Data type	Datasets used	Covering periods	Data sources
Gridded maps	GlobeLand30	2000, 2010, 2020	Chen et al [3]; Jun et al [4]
	Gong's data	1978-2017	Gong et al [5]
	MODIS MCD12Q1	2001–2019	The National Aeronautics and Space Administration
	ESA CCI	1992–2018	European Space Agency
	Global Cropland 30m (Globalcrop30m)	2015	Global Food Security Analysis-Support Data at 30 Meters (GFSAD30) Project
	IGBP Data and Information System	1992–1993	Loveland et al [6]
	UMD Land Cover	1992–1993	Hansen et al [7]
	GLC2000	2000	Bartholomé and Belward [8]
	CAS1990	1980s–1990s	Ran and Li [9]
	WESTDC Land Cover Product v2.0	2000	Ran [10]
	Vegetation map	1980s	CCVM*
	CNLUCC*	2018, 2015, 2010, 2008, 2005, 2000, 1995, 1980s	RESDC***
	FROM-GLC	2010, 2015, 2017	Gong et al [11]
	Yang's cropland	1887, 1933, 1952	Yang et al [12]
	HansenForest	2000-2019	Hansen et al [13]
	LiForest	2010	Li et al [14]
Vector map	1Mveg	1980s	1:1,000,000 vegetation produced by CCVM**

	1Mgrass	1980s	RESDC
Tabular data	National Land and Resources Bulletin (NLRB)	2007–2017	The Ministry of Land and Resources of China
	China Land and Resources Statistical Yearbook (CLRSY)	1999–2001	The Ministry of Land and Resources of China
	China Agricultural Yearbook (CAY)	1981–2018	National Bureau of Statistics of China
	Chinese Statistical Yearbook (CSY)	1949–2016	National Bureau of Statistics of China
	The 1st to the 9th NFI****	1976-2018	The State Forestry Administration of China
	NFGDC*****	1949,1962	The State Forestry Administration of China
	Niu's wetland	1978, 1990, 2000, 2008	Niu et al [15]

*CNLUCC: China Land Use and Cover Change; **CCVM: The Compiling Committee of the Vegetation Maps of China; ***RESDC: The Data Center for Resources and Environmental Sciences, Chinese Academy of Sciences (RESDC) (http://www/resdc.cn); ****NFI: National forest inventory; *****NFGDC: National Forestry and Grassland Data Center (http://www.cfsdc.org/).

Table S2. Datasets used for reconstruction of each land cover type*

Land cover types	Datasets used
Lake, river, barren land**	GlobeLand30
Impervious land	Gong's data
Cropland	MODIS MCD12Q1
	ESA CCI
	Global Cropland 30m (Globalcrop30m)
	IGBP Data and Information System
	UMD Land Cover
	GLC2000

	CAS1990
	WESTDC Land Cover Product v2.0
	1Mveg
	CNLUCC
	FROM-GLC
	GlobeLand30
	Yang's cropland
	National Land and Resources Bulletin (NLRB)
	China Land and Resources Statistical Yearbook (CLRSY)
	China Agricultural Yearbook (CAY)
	Chinese Statistical Yearbook (CSY)
Forest	GlobeLand30
	MODIS MCD12Q1
	ESA CCI
	IGBP Data and Information System
	UMD Land Cover
	GLC2000
	CAS1990
	WESTDC Land Cover Product v2.0
	1Mveg
	CNLUCC
	FROM-GLC
	HansenForest
	LiForest
	The 1st to the 9th NFIs
	NFGDC

Wetland	MODIS FAOLCCS3
	ESA CCI
	IGBP Data and Information System
	UMD Land Cover
	GLC2000
	CAS1990
	CNLUCC
	Niu's wetland
Grassland, Shrubland	1Mgrass
	Reconstructed land cover data*

*Reconstructed land cover data are other land cover types reconstructed in this study, including forest, cropland, wetland, impervious land, river, lake, and barren lands. Abbreviations are same as in the Table S1; **The lake, river, and barren land are fixed maps, while the proportions of other land cover types in each grid-cell change annually.

1.2 Reconstruction of forest data

Before reconstructing forest distributions in China, the composition of forests should be clarified. China's forests can be divided into natural (NF) and planted (PF). The PFs are consisted of three components, viz. forest stands, economic forest, and bamboo forest. In this study, the three components were represented by two different types in model simulations (17 types for forest stands and 1 type for economic and bamboo forests, see Table S4), and the annual composition ratios of each component were determined. Therefore, forest reconstruction was divided into two steps in this study.

First, we determined the total forest and PF areas at national and provincial levels using historical national forest inventories (NFIs), annual yearbooks from the National Forestry and Grassland Data Center (NFGDC), and publications (Table S3). Then, the NF area can be calculated by deducting PF area from the total forest area at each level. Note that PF areas were not available before 1976, thus we extrapolated the PF areas for the period 1900-1976. More specifically, using available PF area data collected from 1976 to 2018, we extrapolated PF back to 1900 using an exponential model (Figure S1a,

R^2=0.997). Here, PFs were divided into forest stands and other PFs (i.e. economic forest and bamboo forest). Forest stands were planted for timber and/or environmental protection purpose. However, such purpose was less required than livelihood-support demands (e.g. food production and economic benefits) in the early period, which were mainly exploited from economic forests and bamboo forests. Thus, we assumed that PF was dominated by economic forests and bamboo forests in 1900 and extrapolated the area using available data (Figure S1b, R^2=0.995). The areas of forest stands were the differences between total PF and the areas of other PFs.

Second, the forest areas were allocated spatially using a model developed previously and successfully implemented in the reconstruction of the cropland distributions in the U.S. and China [1,2] as well as the planted forest area, age, and type in China [16]. Similar to the former studies, the reconstruction processes can be divided into thirteen periods with a base year identified from each period (Table S3). The years between base years were linearly interpolated. Note that the forest areas of the 1st to the 9th NFIs were inventory-based, which were used as benchmark data for validation of forest area changes. Specifically, the changes of forest area from 1900 to each of the base years since 1976 were compared (see main text Fig. 1d).

The spatial distributions of the forests reconstructed are illustrated in Figures S2&S3. We then validated total forest areas and PF areas at provincial level in each baseline years since 1949 (Figure S4&S5). The results showed that our reconstructed forest areas are highly consistent to the provincial NFI data (R^2 = 0.99, Figures S4&S5).

Table S3. Reconstruction of forest data

Periods	Covering years	Base year	Data sources
1	2014-2018	2018	The 9th NFI*
2	2009-2013	2013	The 8th NFI
3	2004-2008	2008	The 7th NFI
4	1999-2003	2003	The 6th NFI
5	1994-1998	1998	The 5th NFI
6	1989-1993	1993	The 4th NFI

7	1984-1988	1988	The 3rd NFI
8	1977-1981	1981	The 2nd NFI; Liu et al [17]
9	1963-1976	1976	The 1st NFI; Liu et al [17]
10	1950-1962	1962	NFGDC**
11	1949	1949	NFGDC
12	1933-1948	1933	Yang and Jin [12]
13	1900-1932	1900	Yang and Jin [12]

*NFI: National forest inventory; **NFGDC: National Forestry and Grassland Data Center (http://www.cfsdc.org/).

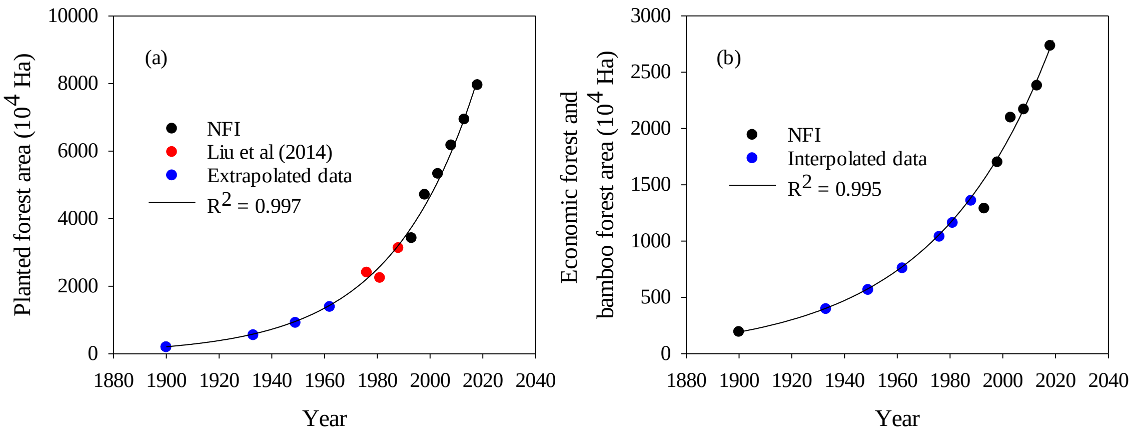

Figure S1. Extrapolation and interpolation of the areas of (a) total planted forest and (b) other planted forests in China (NFI: national forest inventory data; other planted forests: economic forest and bamboo forest)

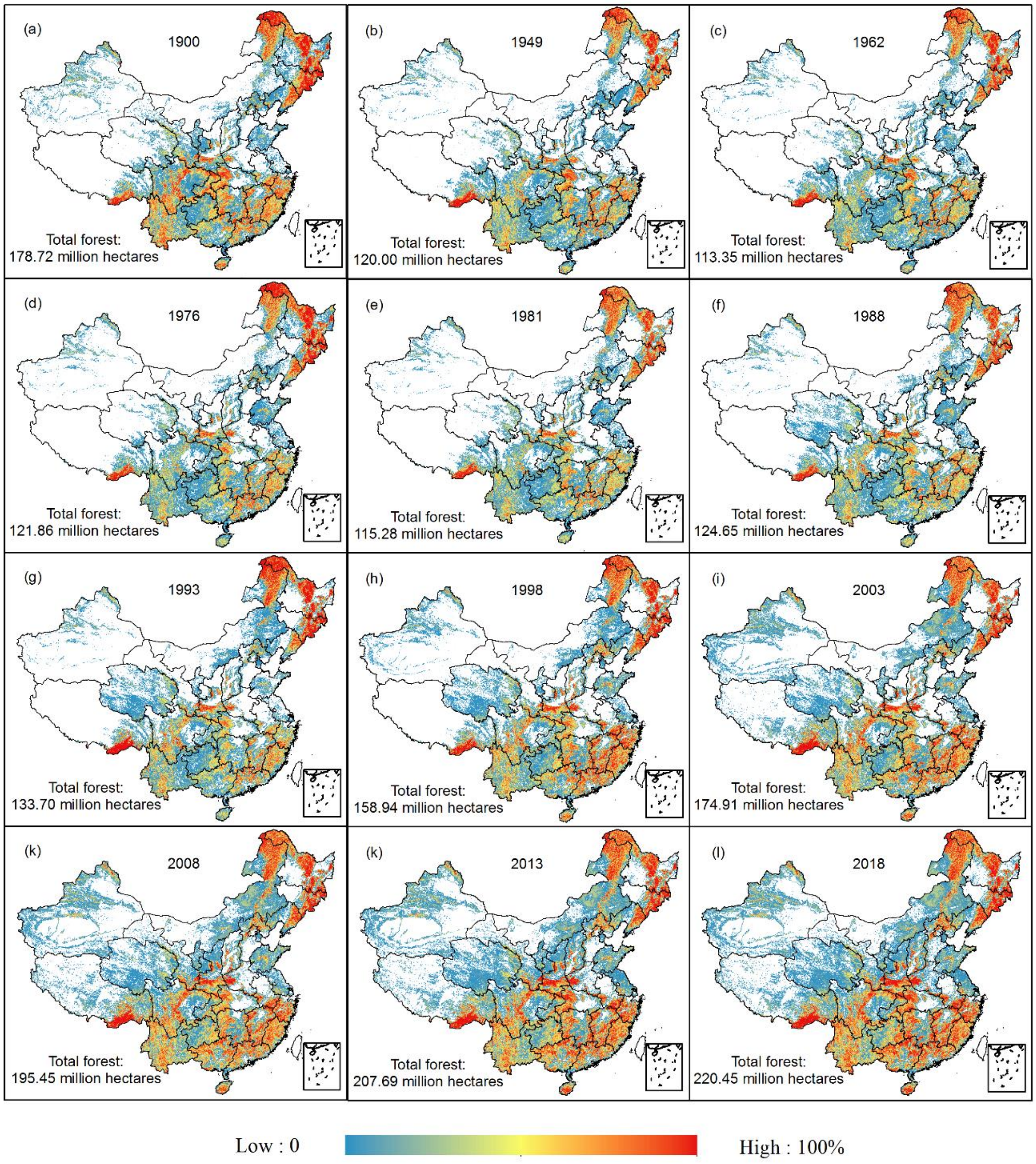

Figure S2. Reconstructed forest distribution across China

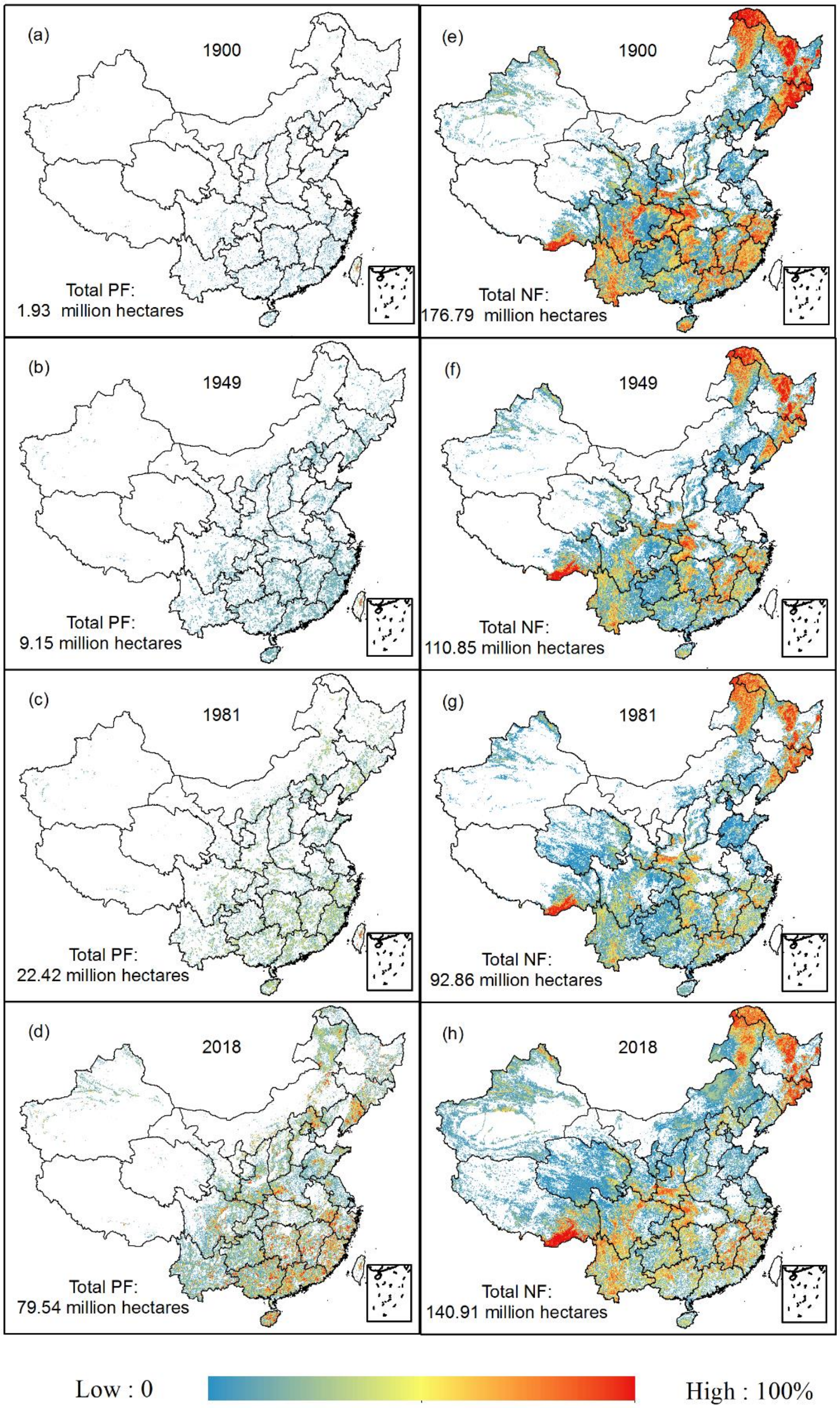

Figure S3. Reconstructed planted and natural forests in China (left panel: planted forest; right panel: natural forest)

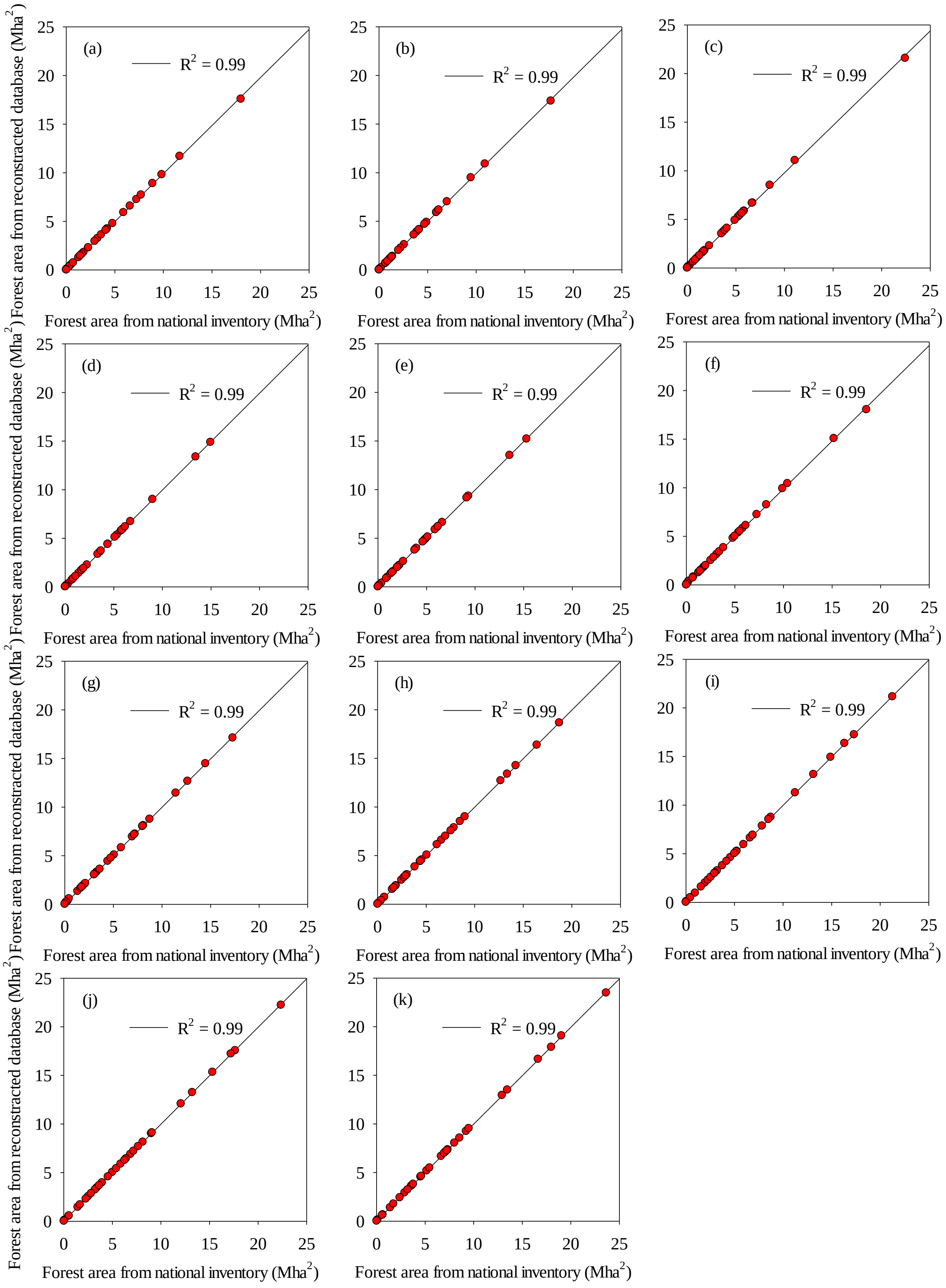

Figure S4. Comparison of the forest areas obtained from reconstructed database and national inventory data at provincial level (Fig. a-k represent the years 1949, 1962, 1976,

1981, 1988, 1993, 1998, 2003, 2008, 2013, and 2018, respectively; the inventory data were provided by State Forestry Administration of China)

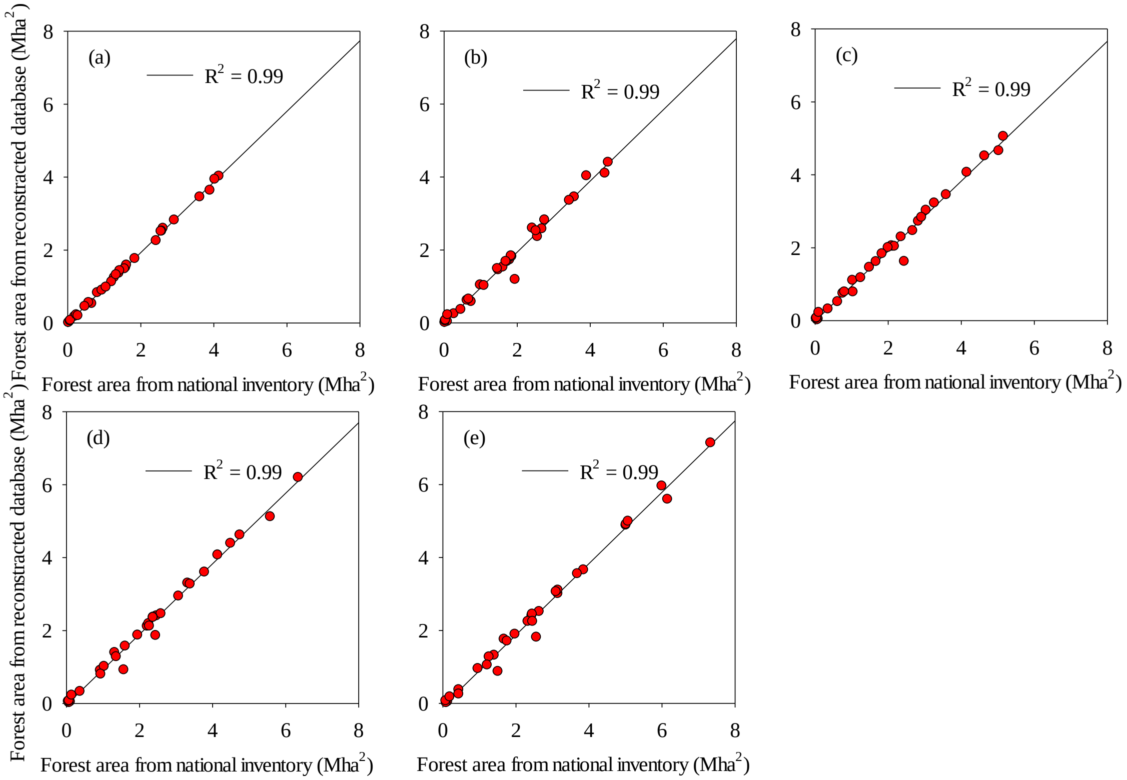

Figure S5. Comparison of the planted forest areas obtained from reconstructed database and national inventory data at provincial level (Fig. a-e represent the years 1995, 2000, 2005, 2010, and 2019, respectively; the inventory data were provided by State Forestry Administration of China)

1.3 Definitions of forest

Forest has different components with specific definitions (Table S4). In FAO Forest Resources Assessment 2015 [18], Chinese forest include 100% of arbor forest, bamboo forest, open forest land, unestablished afforestation land, nursery land, cut-over area & fired-over area, and 73% of economic forest (Table S5; obtained from http://faostat.fao.org/). In comparison, our reconstructed forest dataset includes arbor forest, economic forest, and bamboo forest as previously defined. Therefore, our reconstructed dataset is a "land cover" definition, in which the data was developed to characterize the distribution of annually forested lands. While FAO forest area is more of a "land use" definition, which describes lands have been forested and will continue to be use for forestation (e.g. cut-over area, fired-over area, unestablished afforestation land) (Table S5).

Table S4 Classification and definitions of forest components*

**National class**	**Definition**
Arbor forest	Forest land of arbor species spanning more than 0.0667 ha with a canopy cover of more than 20%.
Economic forest	Forest land of economic species spanning more than 0.0667 ha with canopy cover of more than 20%. The mainly purpose is the provision of non-wooded forest products and fruit.
Bamboo forest	Forest land spanning more than 0.0667 ha, growing bamboo species with the diameter at breast height over 2 cm.
Open forest land	Land of arbor species with canopy cover of which is between 0.10 and 0.19, and 0.0667 ha in size.
Shrub land	Area spanning more than 0.0667 ha with canopy cover of which less than 0.1 and the combined cover of shrub, bushes and tree is more than 30 percent.
Unestablished afforestation land	Areas under afforestation that are temporarily unstocked areas, but can reach the thresholds of forest during 3-5 years; and plot size is

	more than 0.0667 ha
Unestablished enclosure land	Area under enclosure or man-promoted natural regeneration that its regenerated rank reaches middle level and temporarily unstocked but may be forested *in situ*.
Nursery land	Land for cultivating sapling.
Cut-over area & fired-over area	Areas under felling/fired that have not got to above thresholds, but can become forest land in 3 ~ 5 years.
Other non-stocked forestry land	Including: 1) The afforestation area that does not reach the threshold of unestablished afforestation land; 2) Unestablished afforestation land over the threshold of forested time, but not reaching the threshold of forest, shrub and open forest land; 3) Area that is prepared, but temporarily not planted; 4) Forestry land prepared for natural protection, scientific research and forest fire, temporarily not covered by trees, however, it may be covered by scattered trees, shrub and bushes.
Forest suitable land	Area planned for tree planting, including wild land and sandy land liable to forest cultivation. Its canopy cover is less than 0.1, and it is characterized by a combined cover of shrub, bushes and trees less than 30 percent, however, generally more than 10 percent.
Land used for forestry auxiliary production	Area of engineer facilities with forestry land ownership and growing scattered trees and shrub, including forest roads and facilities sites for forestry production.

* Obtained from FAOSTAT (2015) (Available at http://faostat.fao.org/)

Table S5 Forest components adopted in FAO FRA assessments and this study

**National class**	**FAO statistics***	**This study**
Arbor forest	100%	100%

Economic forest	73%	100%
Bamboo forest	100%	100%
Open forest land	100%	
Shrub land		
Unestablished afforestation land	100%	
Unestablished enclosure land		
Nursery land	100%	
Cut-over area & fired-over area	100%	
Other non-stocked forestry land		
Forest suitable land		
Land used for forestry auxiliary production		

* Obtained from FAOSTAT (2015) (Available at http://faostat.fao.org/)

1.4 Reconstruction of wetland, grassland, shrubland, and impervious land data

The spatial distributions of impervious land and wetland were developed separately and independently for the period 1978-2019. The impervious land was directly resampled from Gong et al [5]. For wetland, we allocated the distribution spatially using a similar approach to the one described previously (see Supplementary information 1.1). To do so, we used a wetland map obtained from CNLUCC (Table S1) as a base map, and applied the wetland change information provided by Niu et al [15] to generate the annual wetland maps for the period 1978-2019. The wetland before 1978 and the grassland and shrubland were proportionally adjusted according to the annual dynamics of other land cover types, such as impervious land, cropland, forest, and wetland. Note that the reconstructed database depicts land cover changes. For example, the grassland and shrubland types include land used for forest planting but with no forest formed yet. Therefore, the decline of grassland was majorly from afforestation/reforestation on non-forest land (land used for forest but no forest formed yet). The temporal changes of different land cover types are illustrated in Figure S6.

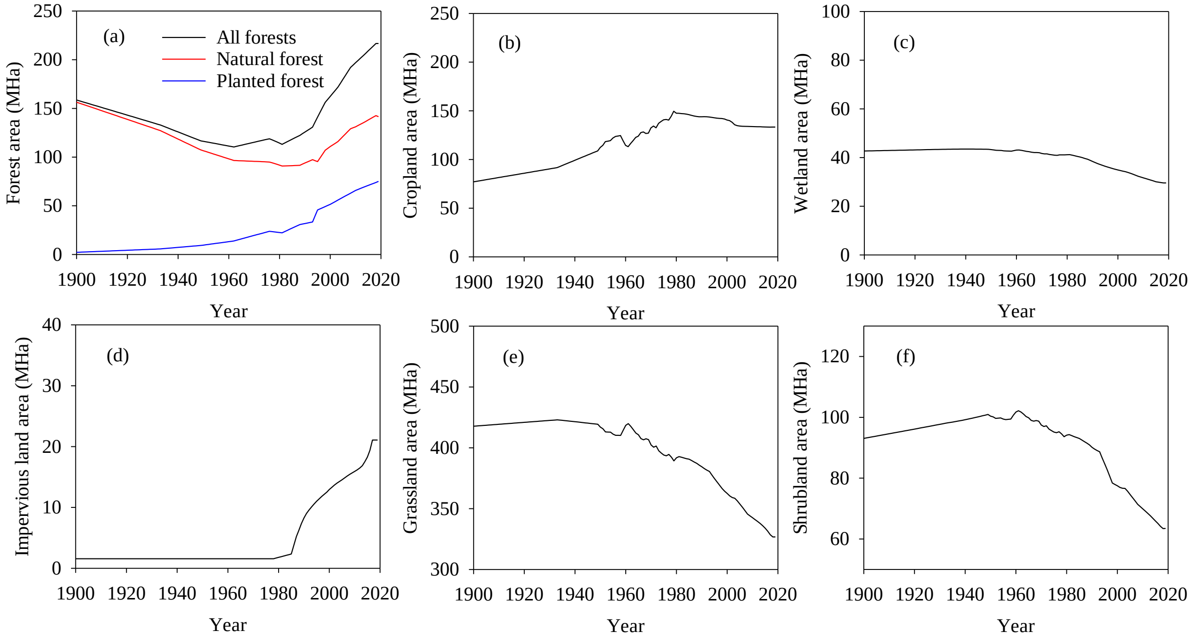

Figure S6. Areas of different land-cover types reconstructed in China from 1900 to 2019 (Fig. a-f indicate forest, cropland, wetland, impervious land, grassland, and shrubland)

2. Comparison of LUCC data used in different studies

The LUCC forcing data used in MsTMIP (LUH_M) and TRENDY (LUH_T) simulations were both developed heavily relying on land-use harmonization (LUH2) data, which therefore inherited the footprint of the LUH2 data and were similar in the temporal changes (Figure 7). In MsTMIP, the LUCC forcing dataset was developed by merging a static satellite-based land cover product, SYNergetic land cover MAP (SYNMAP) [19], with the time-varying LUH2 data for the fifth Assessment Report (AR5) of the Intergovernmental Panel on Climate Change (IPCC) [20,21]. Note that LUH_T dataset and TRENDY (v9) simulations used in this study have been released (https://sites.exeter.ac.uk/trendy/), and a new version (TRENDY v10) is in preparation using an updated LUH2 data version (https://daac.ornl.gov/VEGETATION/guides/LUH2_GCB2019.html). However, we compared the two LUH2 datasets and the differences were trivial in China. Therefore, only the LUH_T used in TRENDY (v9) simulations are presented here.

The LUH_T was directly obtained from LUH2 (v2h) for the period before 1950, while the later period was adjusted using the most recent FAO release until 2012 and extrapolated to 2019 (http://sites.exeter.ac.uk/trendy/protocol/). More specifically, LUCC data of the period 1900-1950 are the same with LUH2 v2h (released for CMIP6). For this period, LUH2 (v2h) was derived from History database of the Global Environment (HYDE) data, based on a previous FAO release that included data up to 2012. The new data from HYDE, prepared for Global Carbon Budget 2019, are based on the most recent FAO release, which includes data up to 2015 (HYDE applied annual changes in FAO data to the 2012 data from the previous release to get the new 2013-2015 data used for Global Carbon Budget 2019). After 2015, HYDE extrapolates the cropland, pasture, and urban data, based on a moving window of the previous 5 years, to generate data until 2019.

Thus, the land-use forcing datasets in MsTMIP and TRENDY were developed using similar drivers at a global extent. These datasets are advantageous in the global and long-term coverage as they were designed for use in a consistent global framework. We compared the three datasets and found that the LUH_M and LUH_T are greatly biased in representing the status and variations of the major biomes' areas in China (e.g. forest,

cropland) (Figure S7). In general, the forest areas in LUH_M and LUH_T are consistent before 1950, while forest area declines faster in LUH_T after 1950 (Figure S7a). Moreover, the disagreement of cropland areas begins in the early 1980s between the two datasets (Figure S7b). Our newly reconstructed and validated LUCC data revealed that forest area stagnated from 1950 to 1980 and rapidly recovered thereafter, while the cropland area shrunk after the 1980s (Figure S7b).

Note that the HILDA+ data were harmonized from FAO-statistics and remote sensing images [22]. Similar to our study, the potential allocation of land cover change was based on probability maps derived from gridded images in HILDA+. Different from our model, the trigger of allocations in spatial were limited by threholds predetermined in reconstructing the HILDA+ database. During the reconstruction process, the 2015 map served as a starting point, and the model run backward (2015-1960) and then forward (2015-2019) in time [22]. Therefore, we suspect that the large change in FAO-cropland statistics (e.g. cropland changes, Figure S7) was damped (reduced) by the limiting threshold applied, resulting into a minor cropland decrease from the 1980s to the mid-1970s (Figure S7b). Similarly, when rebuilding cropland maps backward from the mid-1970s to 1960, the slow changes of cropland in FAO-statistics were fully inherited by the HILDA+ database, causing a large bias (overestimation) of cropland arceage in the 1960s (i.e. intensive cropland abandoment from 1960 to 1980 in Figure S7b).

We also performed spatial comparisons of the reconstructed cropland and forest with existing databases and remote-sensing-based products in the years 1980, 1990, and 2018 (Figure S8&S9). Our reconstructed maps are more consistent in depicting forest and cropland distribution if the remote-sensing images were used as a reference.

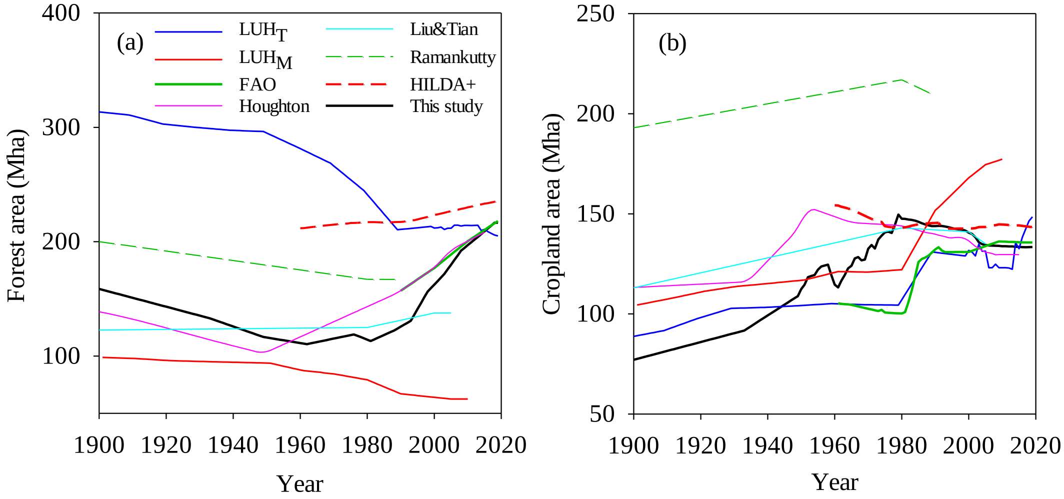

Figure S7. Historical changes of (a) forest and (b) cropland from 1900 to 2019 obtained from different datasets (unit: Mha). (Houghton data were derived from Houghton and Nassikas [23] and the data in 1900 were interpolated from 1850 and 1950; Liu&Tian and Ramankutty were derived from Liu and Tian [24] and Ramankutty and Foley [25], respectively; HILDA+ was from Winkler et al [22])

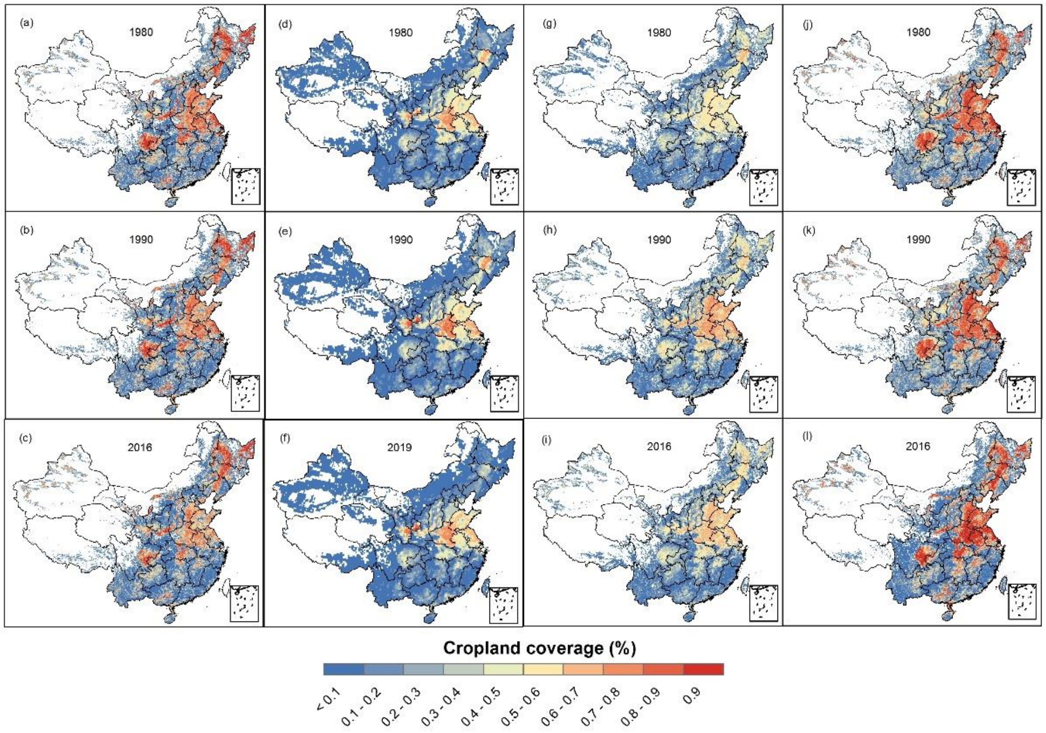

Figure S8. Comparison of the cropland distributions from (a, b, c) this study, (d, e, f) LUH-GCB, (g, h, i) HYDE, and (j, k, l) remote sensing-based images since 1980. (the remote-sensing based images were from CNLUCC derived from Landsat data)

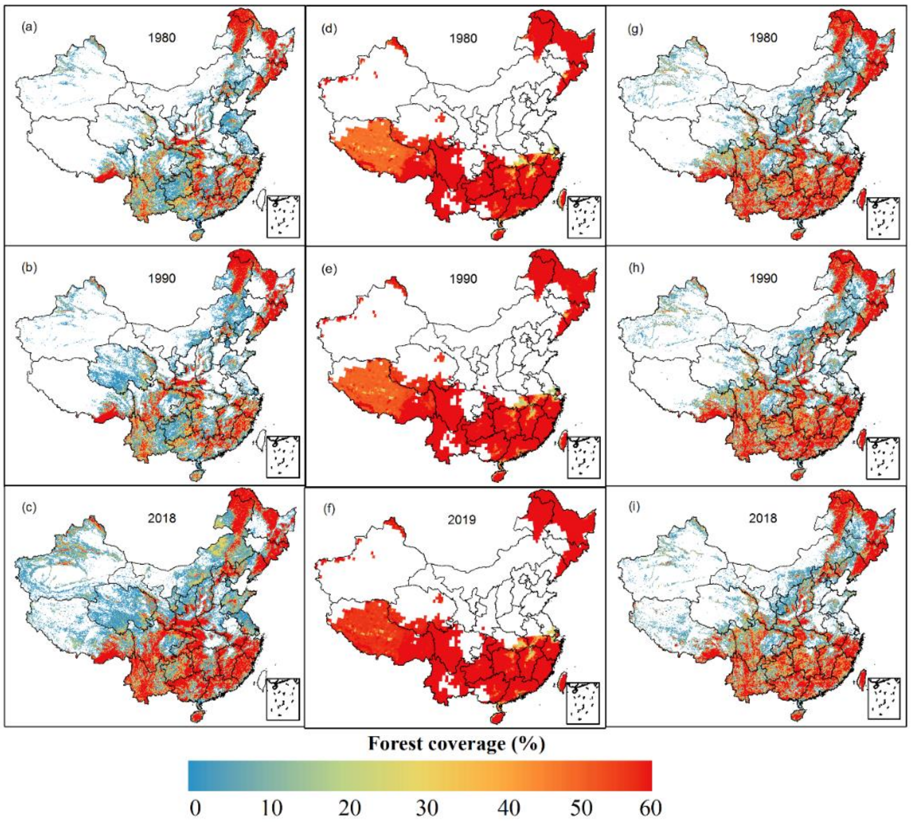

Figure S9. Comparison of the forest distributions from (a, b, c) this study, (d, e, f) LUH-GCB, and (g, h, i) remote sensing-based images since 1980. (the remote-sensing based images were from CNLUCC derived from Landsat data)

3. Biases in FAO and LUH2 cropland datasets

Two abrupt increments were observed from former LUCC datasets. The first one is observed during the period of 1980 to 1990 (abnormal increment of 28–32 Mha) in FAO and LUH2 (i.e. TRENDY, MsTMIP in Figure S10, shaded in grey), which highly differs from the 4 Mha decline revealed with our reconstructed data. This is because the official data reported by FAO were from the China Agricultural Yearbook (CAY) whose cropland underestimations are now officially acknowledged. For the period from 1982 to 2007, FAO received data reported from the China Land and Resources Statistical Yearbook (CLRSY), resulting in an abrupt change in the cropland acreage (Figure S10).

The second abrupt increment was found in the period of 2007 to 2010 (Figure S10, shaded in grey), in which the cropland areas reported to FAO shifted again from CLRSY to the National Land and Resources Bulletin (NLRB). Such shift was also because of the advancing technologies adopted in cropland surveying (e.g. higher resolution satellite images than previous period, drones etc), by which the small, fragmented croplands were able to be identified. Therefore, similar abrupt increases in the cropland in LUH2 was inherited from FAO, which was used as the basis for the LUH2 reconstruction. In comparison, our newly developed cropland data corrected such errors (black line in Figure S10).

It should be noted that the most recently updated FAO statistics in 2021 corrected the abrupt cropland change from 2005 to 2010 (i.e. FAO-new in Figure S10). However, due to the large underestimations of cropland area in 1980 to 1990s were not corrected, the cropland abandonment from 1990 to 2010 was still misrepresented as expansion in FAO-new data (Figure S10). Thus, FAO-statistics should be used cautiously for reconstructing LUCC data in China.

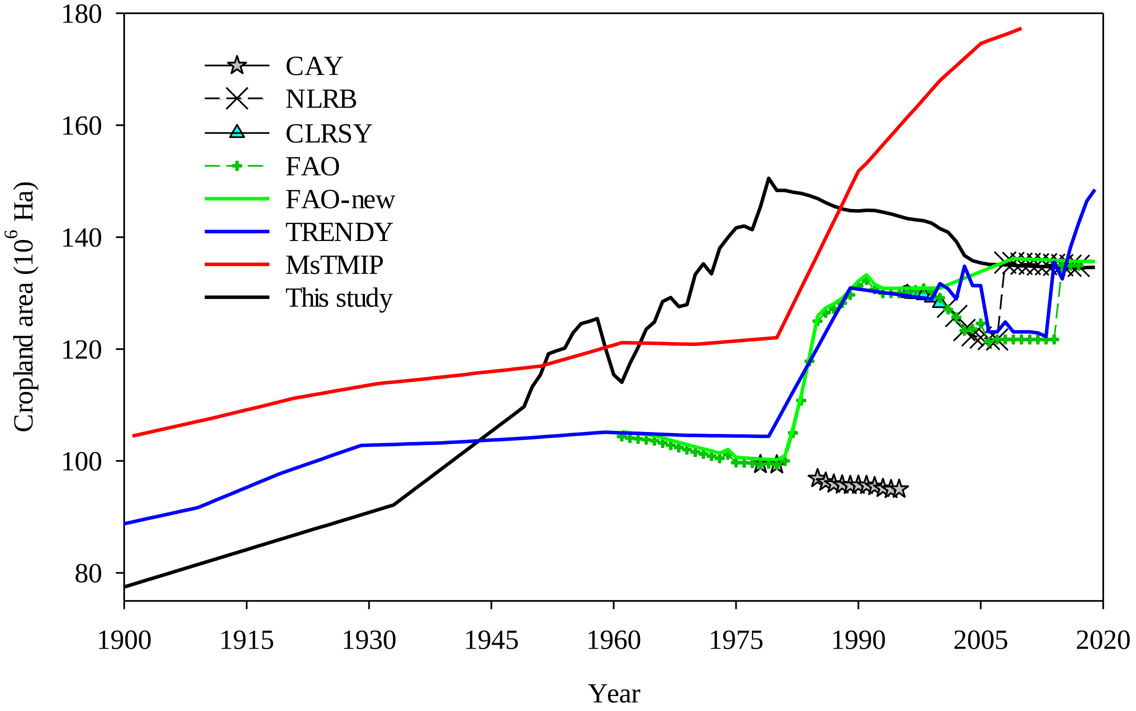

Figure S10. Comparisons of China's cropland area data from different sources. The abbreviations used in the figure are as follows: CAY: Chinese Agricultural Yearbook; NLRB refers to the National Land and Resources Bulletin; CLRSY denotes the China Land and Resources Statistical Yearbook; FAO: cropland data provided by Food and Agriculture Organization of the United Nations in 2020; FAO-new: new cropland area updated by FAO in 2021; The two abrupt cropland increments in previous LUCC databases were shaded in grey.

4. Field measured sites used

Here we used data obtained from field measurement to calibrate and validate the DLEM model. The field-survey data were collected from a nationwide field campaign in China's forests. Based on our former studies [1,16], we divided forests into 18 types, including *Pinus massoniana*, *Cunninghamia lanceolate*, *Eucalyptus* spp., *Populus* spp., *Larix* spp., *Pinus tabulae*, Cypress, *Pinus elliottii*, *Pinus sylvestris*, other Pinaceae species, broadleaf mixed forests, deciduous broadleaf forests, evergreen broadleaf forests, needle-leaf and broadleaf mixed forests, needle-leaf mixed forests, *Quercus* spp., *Picea* spp., *Abies* spp., and economic and bamboo forests (Table S6). For each forest type, we also randomly selected undisturbed sites from the national field campaign to calibrate and validate the DLEM model (Figure S11).

Table S6. The 18 forest types used in model simulations.

ID	Forest type
1	*Pinus massoniana*
2	*Cunninghamia lanceolata*
3	*Eucalyptus* spp.
4	*Populus* spp.
5	*Larix* spp.
6	*Pinus tabulae*
7	Cypress
8	*Pinus elliottii*
9	*Pinus sylvestris*
10	Other Pinaceae species
11	Broadleaf mixed forests
12	Deciduous broadleaf forests
13	Evergreen broadleaf forests
14	Needle-leaf and broadleaf mixed forests
15	Needle-leaf mixed forests
16	*Quercus* spp.
17	*Picea* spp.; *Abies* spp.
18	Economic and bamboo forests

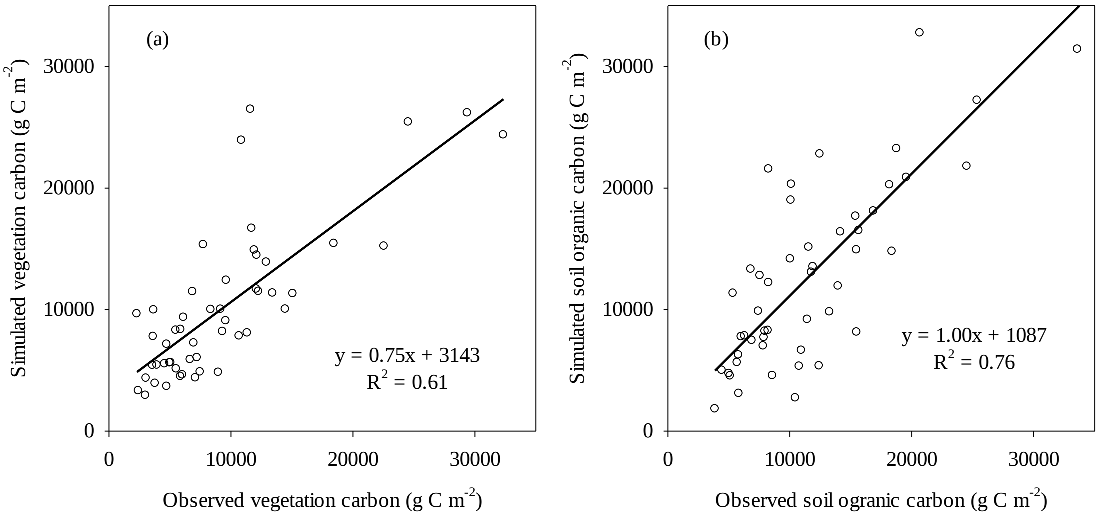

Figure S11. The model calibration using measured data from the national field data survey (a: vegetation biomass carbon; b: soil organic carbon at 0-100 cm depth)

5. Forest carbon loss from harvesting

Forest harvesting was separated into industrial wood harvest and fuel wood harvest. The proportions of harvesting from industrial wood and fuel wood were obtained from Houghton and Nassikas [23]. For fuel wood harvesting, all aboveground biomass is burned directly, and the belowground biomass enters into litter pool. For industrial wood harvesting, 50% of the aboveground biomass enters litter pool and the rest 50% is distributed into 10- and 100-year wood product pools. Total, annual forest harvest areas were obtained from the LUH2-GCB database, which was further separated into harvesting in NF and PF (Figure S12), respectively. The harvested C loss is showed in Figure S13.

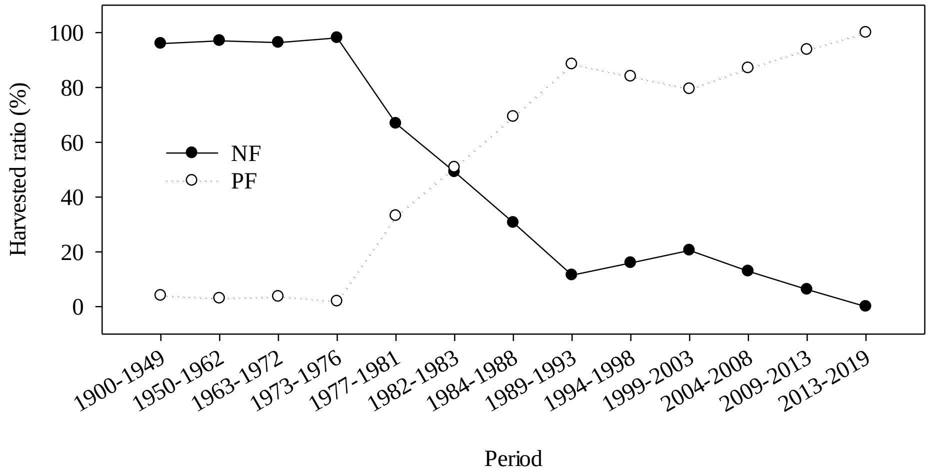

Figure S12. Harvested area ratios from NF and PF from 1900 to 2019. The numbers indicate the proportion of NF or PF harvested in comparison to the total harvested area in each period.

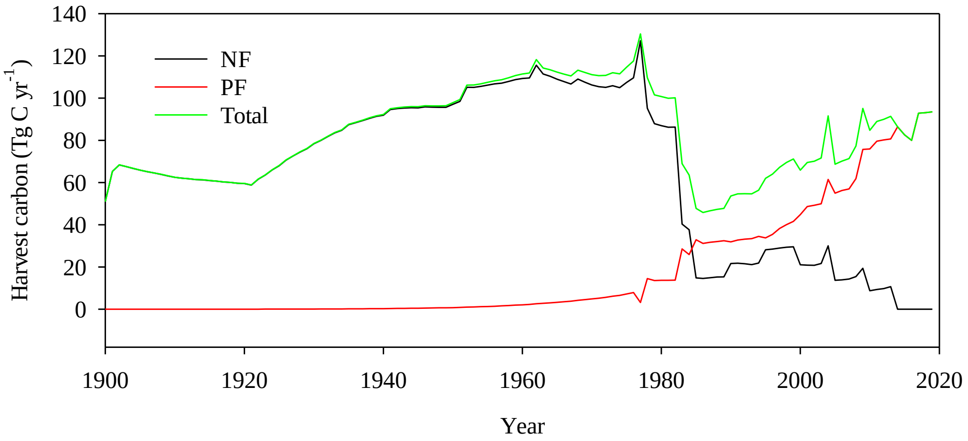

Figure S13. Carbon loss from forest harvesting

6. Validations of the changes in simulated forest carbon stock

We validated our simulated forest C stock changes with inventory-based estimates obtained from various studies at both provincial and national scales (Figure S14). Four provincial and three national estimates were used (Table S7). The provincial forest C storage datasets include estimations from Li et al [26] and Guo et al [27] derived from data collected during the 7th NFI (2004-2008), Wu et al [28] derived from the 4th-6th NFI (1989-2003), and the 9th NFI provided by the National Forestry and Grassland Administration of China. The national forest C storage changes were obtained from Fang et al [29], Zhang et al [30], and Guo et al [27] covering the periods 1950-1998, 1949-2008, and 1984-2008, respectively. Other published estimates in different provinces were also collected, and the average of the C storage was used if multiple estimates were available (Table S7).

Note that different models were used in forest C stock estimations, despite all studies were inventory-based. Besides, the C stock estimated by the National Forestry and Grassland Administration of China [31] did not include open woodland, scarcely forested land, and border trees, while they were included in other datasets in 2003-2008. Since the C stock of these components was minor (i.e. 0.5928 Pg in total from Li et al [26]) and is expected stable, we add this part to the 9th NFI dataset to reduce the biases. The C was allocated to each province by the area reported in the NFI. The validations revealed that our estimations are capable to capture C storage changes at both provincial and national levels (Figure S14).

Table S7. Inventory-based estimates used in validation of forest carbon storage changes*.

References	Periods**/year	Region
Li et al [26]	2003-2008	Provincial estimates
National Forestry and Grassland Administration [31]	2014-2018	Provincial estimates
Wu et al [28]	1990-2000	Provincial estimates
Guo et al [27]	1984-2008	Provincial and national estimates
Zhang et al [30]	1949-2018	National estimates
Fang et al [29]	1950-1998	National estimates
Wang et al [32]	2006	Jilin

Liu et al [33]	2009-2013	Tibet
Li et al [34]	2006	Guizhou
Ma et al [35]	2006	Shaanxi
Zhang et al [36]	2006	Heilongjiang

*Data of Taiwan, Hong Kong, and Macaw were excluded; **each national estimate includes multiple sub-periods (NFIs performed in each sub-period).

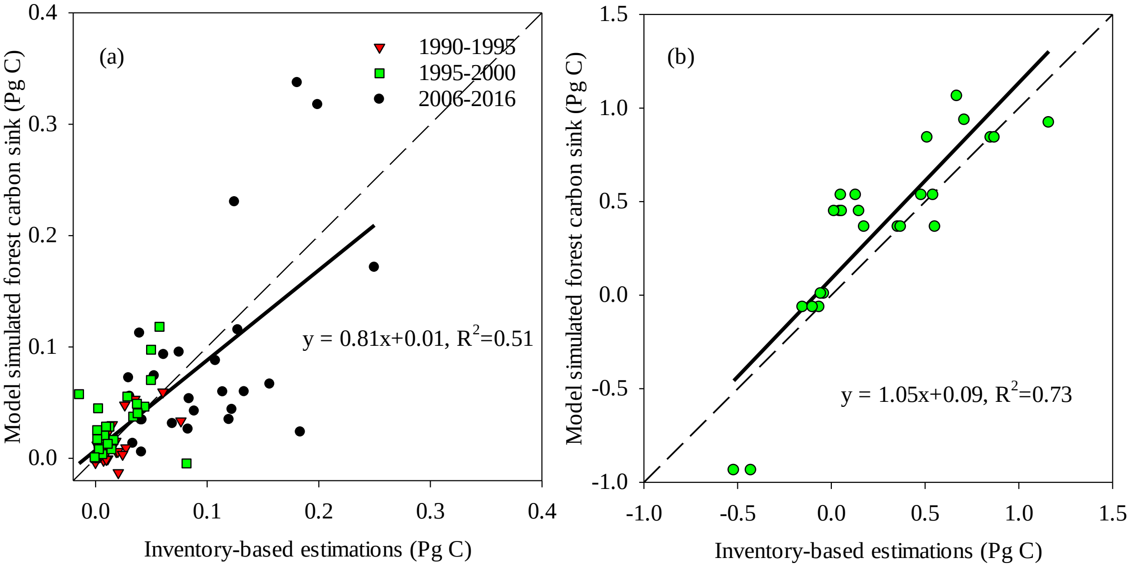

Figure S14. Comparisons of the simulated forest carbon stock change to the inventory-based estimations at (a) provincial and (b) national levels (Fig. a: carbon stock changes for the three periods in each province, viz.1990-1995, 1995-2000, and 2006-2016; Fig. b: carbon stock changes at national level during different sub-periods (NFIs) from 1949-2018)

7. Distribution and changes of simulated carbon stock

Our simulated vegetation C was mainly contributed from forest, grassland, shrubland, and wetland at 9.89±0.42, 1.73±0.002, 0.97±0.002, and 0.28±0.001 Pg C (Table S9), respectively. Vegetation C was mainly distributed in the south and northeast regions, while high SOC was mainly distributed in southwest and northeast regions of China (Figure S15a&d). The distribution pattern of our simulated vegetation C is similar to a remote sensing-based product (Figure S15a&b), in which the total vegetation C stock (excluding Taiwan) was 13.32 Pg C in 2010 [37]. Estimation of vegetation C from the IPCC Tier-1 Global Biomass Carbon Map in 2000 was 30.51 Pg C [38] (Figure S15c), which was obviously overestimated when comparing with both remote sensing- and inventory-based results (e.g. Tang et al [39], Table S9). Our model-simulated vegetation C stock was 12.53±0.63 in the 2010s (Table S9, Figure S15a), and both the spatial distribution pattern and the total estimation are close to the results derived from remote-sensing product (Figure S15b). We also compared our simulated forest biomass C with officially released data from the National Forestry and Grassland Administration of China (Figure S16). The results showed that our simulation can well-capture the spatial distribution of forest C stock in China (Figure S16, R^2=0.75).

Our model-simulated total C stock was 90.06±0.82 Pg in the 2010s, in which SOC was 77.55±0.46 Pg C (Table S9, Figure S17). We compared our estimated SOC in 2019 with other published data, and the results revealed that the general distribution patterns were similar, despite the magnitude differed by locations between studies (Figure S15d-f). Specifically, the machine-learning-based estimation (145 Pg C, Figure S15f) [40] seems to systematically overestimate SOC when comparing national total with inventory-based results (Table S9), while our simulated SOC of 77.55±0.46 Pg C was more close to inventory-based estimations (Table S9). The overestimation is probably due to a limited sample size from China, compared with global samples, in which soil samples from China account to less than 2%, causing underrepresentation of low SOC density in China from using a globally-based machine-learning model [40]. Our simulation may overestimate SOC in the western Tibet Plateau (Figure S15d-e) compared with a newly developed SOC map [41]. However, since the region was barely affected by LUCC, the overestimation is expected to have minor impacts on our historical analyses and attribution analyses. For

model intercomparison results, estimations of vegetation C and SOC from MsTMIP (Figure 17d-f) and TRENDY (Figure 17g-i) were similar, but their magnitude differed spatially. In comparison with the vegetation C (20.96-28.12 Pg C) and SOC (69.34-78.35 Pg C) derived from MsTMIP and TRENDY, our simulations are more close to inventory-based estimates (Table S9).

Estimations of China's forest biomass C stock largely varied among studies, ranging 5.51-11.49 Pg C during the 2000s and the 2010s [26,42–46]. Our model-simulated biomass C stock was 9.89 ± 0.42 Pg C in forest (Table S9), which occurs within the aforementioned range. Based on the most recent studies reported, covering 2011-2015, the total forest C stock is much better constrained between 10.48 and 11.49 Pg C [39,43], which is close to the value of 9.89 ± 0.42 Pg C derived from our simulation. Besides, the total vegetation C of 12.53 ± 0.63 Pg C derived from our simulation is also close to the reported value of 14.29 Pg C, while the SOC was slightly higher (77.55 ± 0.46 Pg C) than the reported value of 74.98 Pg C (Table S9).

We examined the C stock change from 1900 to 2019, and found that SOC changes were relatively small in MsTMIP, TRENDY, and this study (Figure 18b, e, h). Nonetheless, vegetation C stock changes largely differed between this study and the other two model intercomparison projects (Figure 18a, d, g), which determines the total C stock change pattern during that period (Figure 18c, f, i). Both MsTMIP and TRENDY simulations showed that vegetation C was lower in most of the area in 2019 than in 1900, while our simulation revealed that vegetation C was higher in some of the region in 2019 (Figure 18d&g). Similarly, both MsTMIP and TRENDY simulations showed that LUCC has triggered a dramatic C stock loss in China since 1980 (Figure 19d-i). Conversely, our model simulation revealed that LUCC has enhanced C stock in most areas of China from 1980 to 2019, except for a small area in the northeast region (Figure 19a-c).

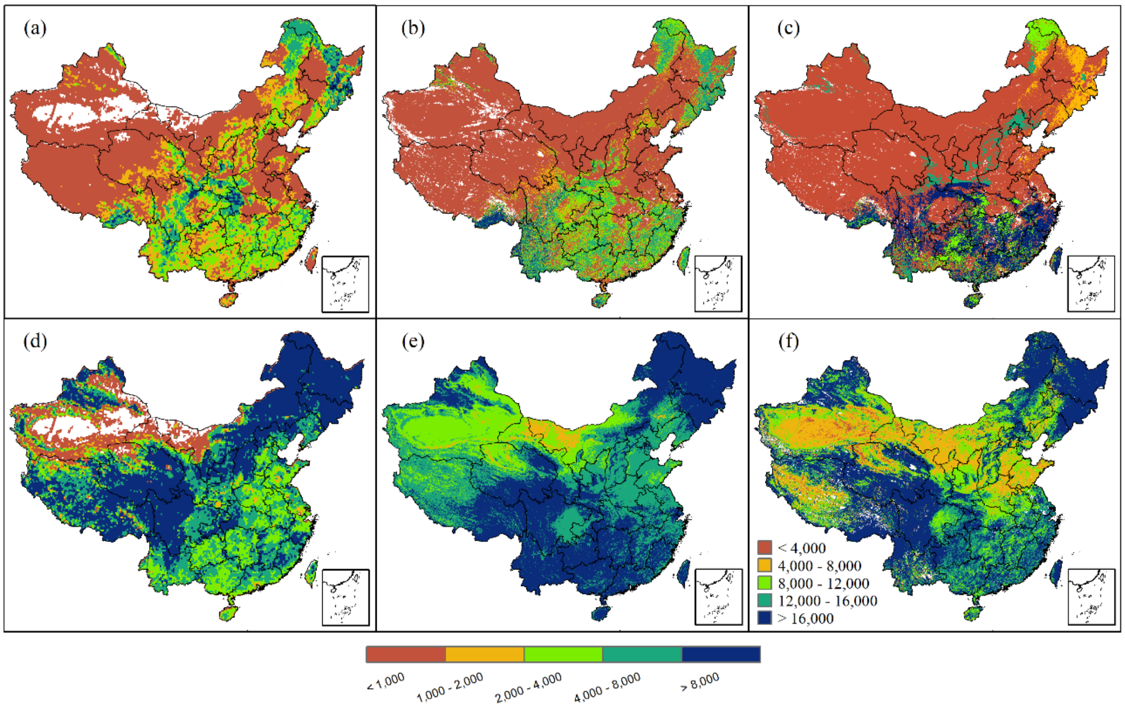

Figure S15. Comparisons of the (a, b, c) vegetation C stock and (d, e, f) SOC in China between our simulated results and other published data (Figures a&d show our simulated results in 2019, Figures b&c show vegetation C obtained from Seth et al [37] and Ruesch and Gibbs [38]; Figures e&f show SOC provided by Liu et al [41] and Hengl et al [40]; note that Figure S15f used a different scale for displaying purpose)

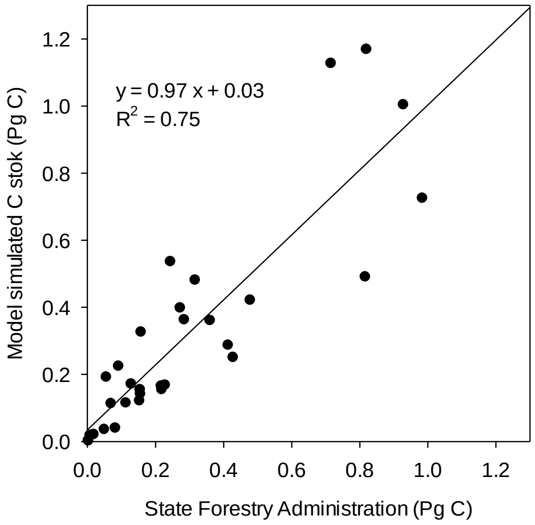

Figure S16. Comparison of the simulated C stock in each province to the data officially released by the National Forestry and Grassland Administration of China in 2019

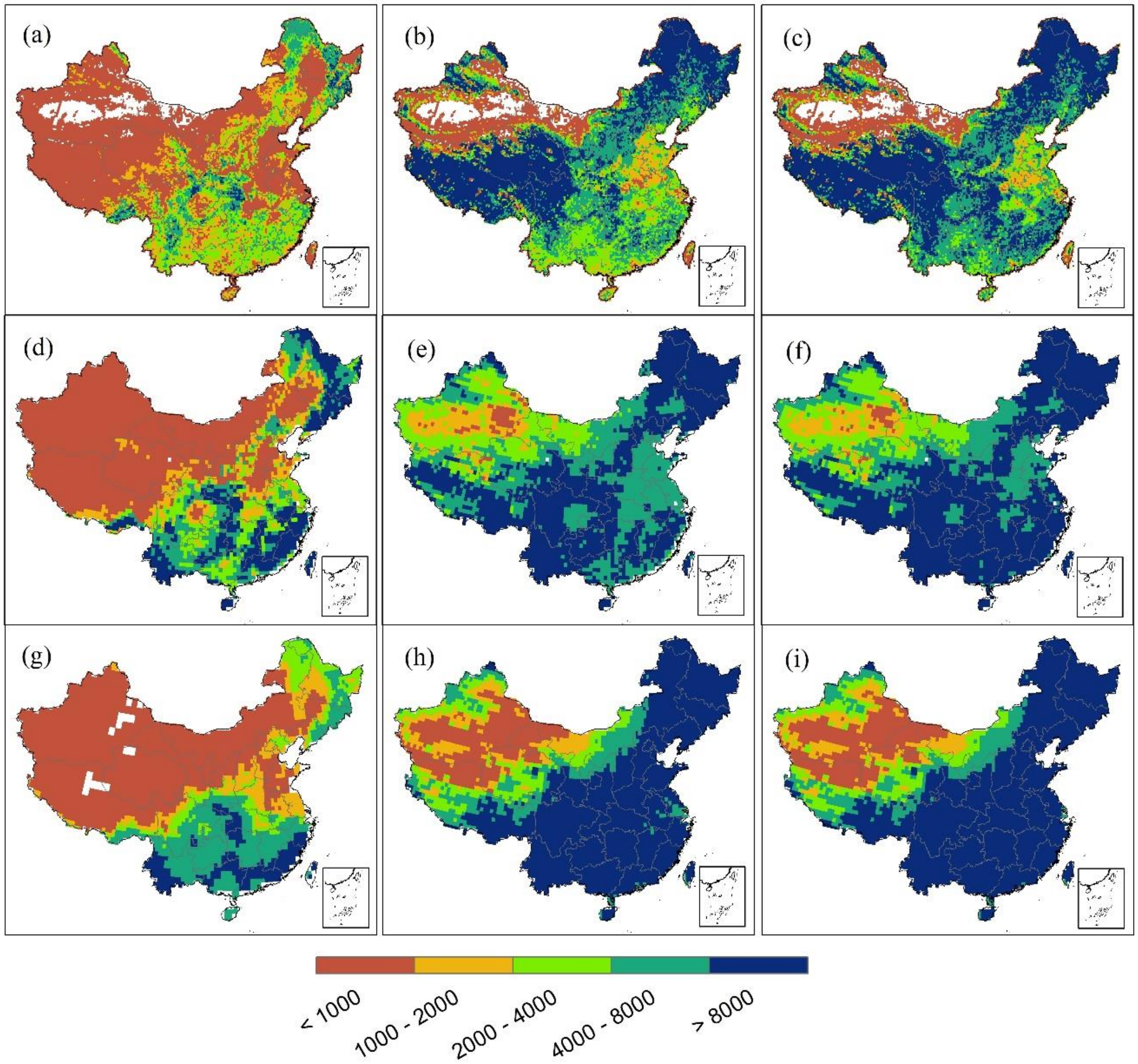

Figure S17. Spatial distribution of carbon stock in China in 2010s from (a, b, c) this study, (d, e, f) MsTMIP, and (g, h, i) TRENDY. (from left to right columns: vegetation carbon, soil organic carbon at 0-100 cm depth, and the total carbon stock; the C stock is the last simulated year in each dataset, which is 2019 for this study and TRENDY and 2010 for MsTMIP)

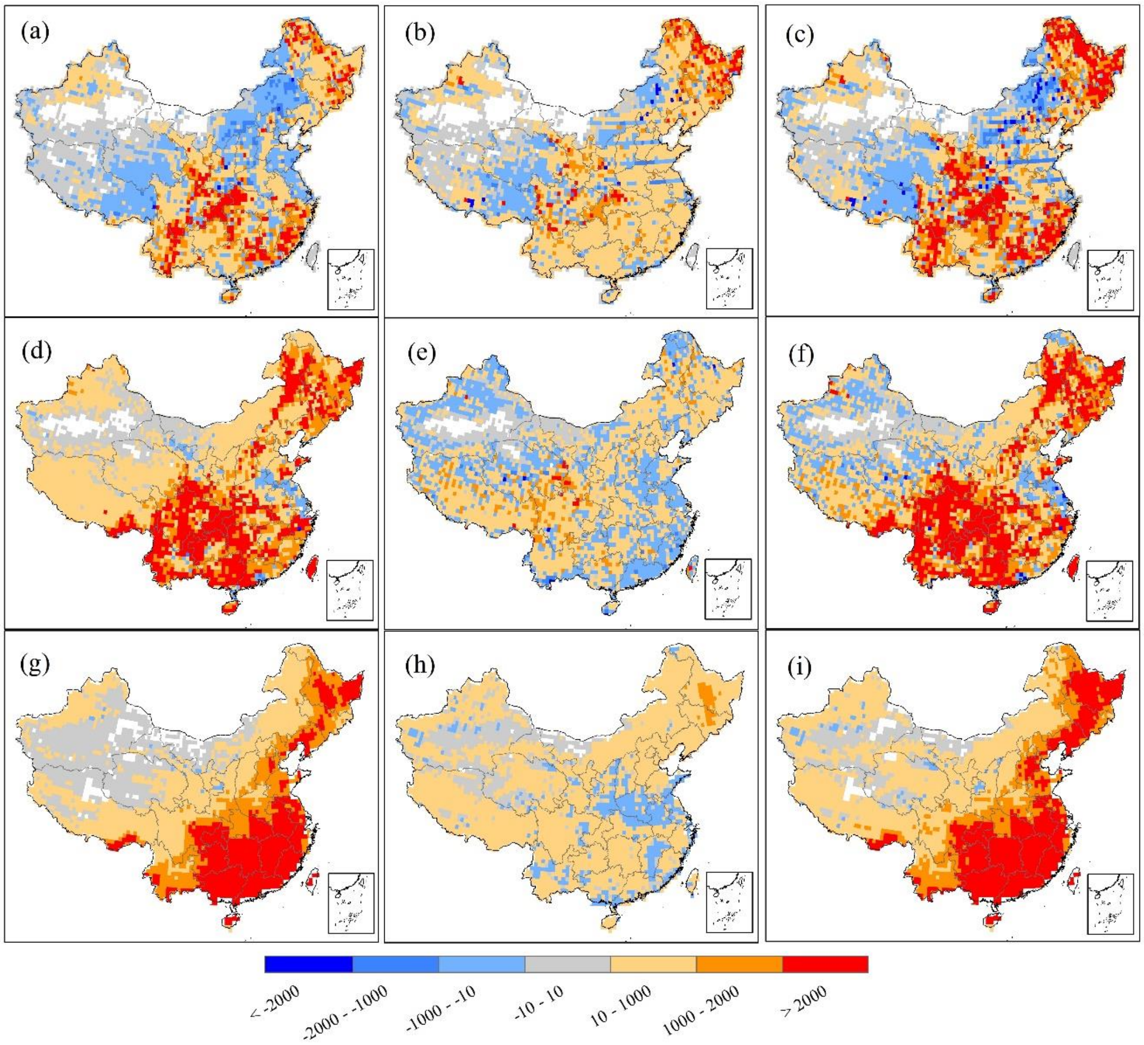

Figure S18. Spatial distribution of LUCC impacts on carbon stock from 1900 to 2019 derived from (a, b, c) this study, (d, e, f) MsTMIP, and (g, h, i) TRENDY (from left to right columns: vegetation carbon, soil organic carbon at 0-100 cm depth, and the total carbon stock; negative and positive value indicate sink and source, respectively; unit: g C m^{-2}).

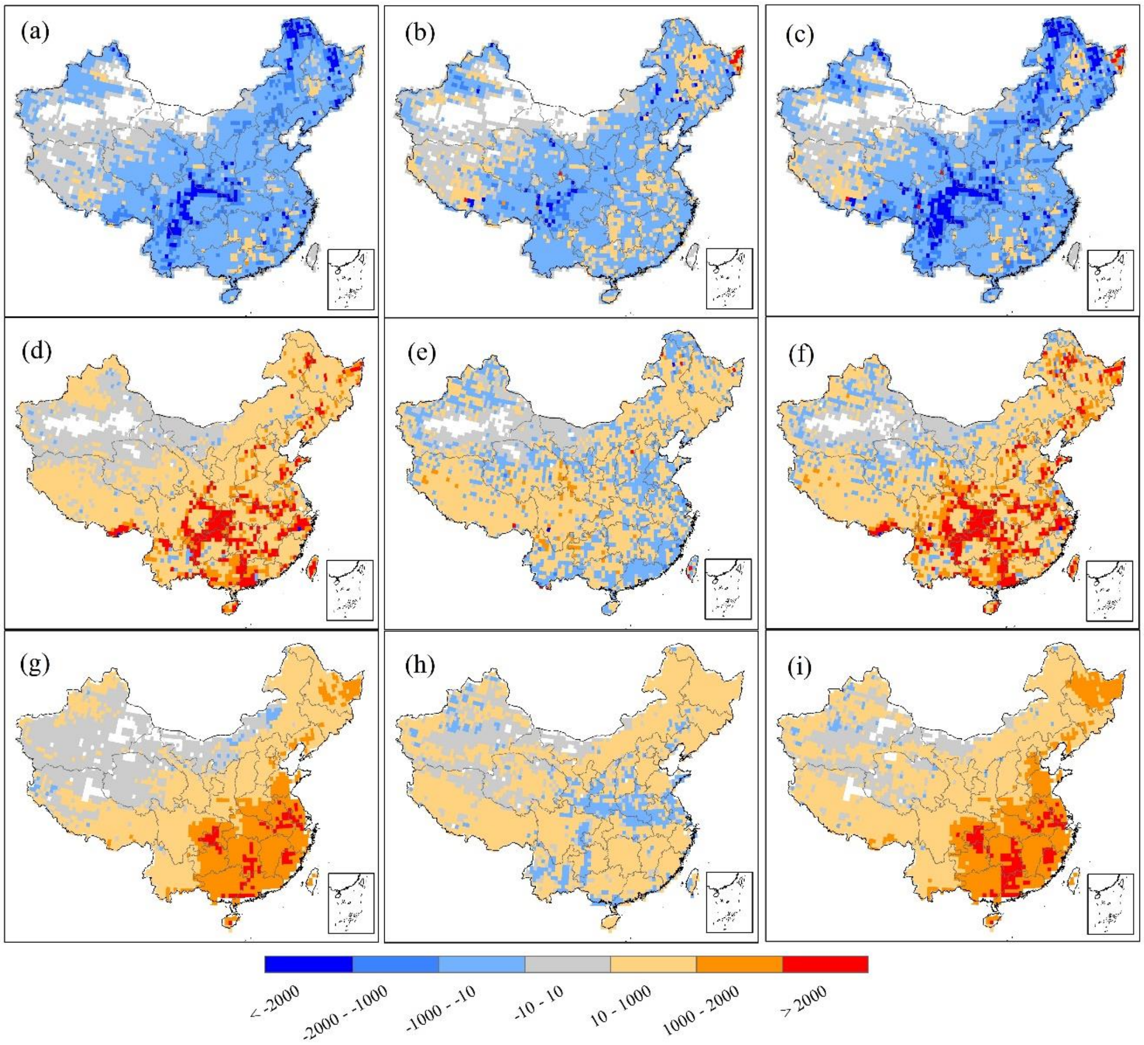

Figure S19. Spatial distribution of LUCC impacts on carbon stock from 1980 to 2019 derived from (a, b, c) this study, (d, e, f) MsTMIP, and (g, h, i) TRENDY (from left to right columns: vegetation carbon, soil organic carbon at 0-100 cm depth, and the total carbon stock; negative and positive value indicate sink and source, respectively; unit: g C m^{-2}).

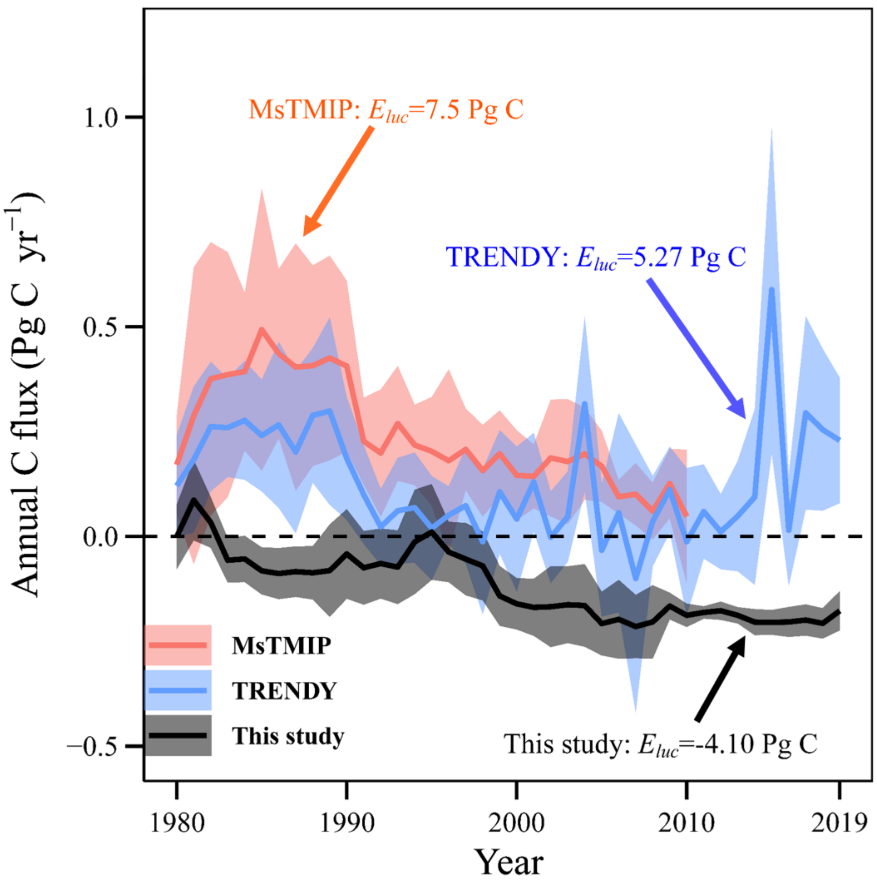

Figure S20. Model estimated annual C fluxes induced by LUCC in China from 1980 to 2010s (E_{luc}: the total C flux induced by LUCC during the period of 1980 to 2010s; positive: C source; negative: C sink; the red, blue and black lines indicate the LUCC-induced C flux derived from MsTMIP (1980-2010), TRENDY (1980-2019), and this study (1980-2019), respectively; the shade areas are the ranges of 1 standard deviation; unit: Pg C; Note that the C fluxes of TRENDY and this study are from 1980 to 2019, while MsTMIP is from 1980 to 2010)

Table S8. Comparison of LUCC-induced carbon fluxes from vegetation and soil in China

Reported C flux (Pg C yr^{-1})[a]			Period	References	This study
Vegetation	Soil	Total			
-	-	0.008-0.05	1990s	Houghton [47]; Houghton and Hackler [48]	-0.056 ±0.041
-	-	-0.03	1990s	Tian et al [49]	-0.056 ±0.041
-0.0132	0.0115	-	1990-2010	Lai et al [50]	[-0.035 ±0.019, -0.087 ±0.068] [b]
-	0.008 [c]	-	1990-2000	Liu et al [51]	-0.030 ±0.020

[a] negative and positive values indicate C sink and source, respectively; [b] vegetation and soil C flux respectively; [c] including soil C change from forest, grassland, and cropland.

Table S9. Comparison of C stocks reported in China

Area* (Mha)	Region /Biome	C stock (Pg C)			Period	Reference/source
		Vegetation	Soil	Total		
946.4	China	14.29 ±0.74	74.98 ±1.28	89.27	2011-2015	Tang et al [39]*
960	China	28.12 ±9.03	78.35 ±27.43	104.99 ±31.29	2010	MsTMIP
960	China	20.96 ±4.76	69.34 ±26.37	90.30 ±28.26	2010s	TRENDY
960	China	12.53 ±0.63	77.55 ±0.46	90.06 ±0.82	2010s	This study
960	China	-	72.5	-	1979-1985	Shangguan et al [52]
188.2	Forest	10.48 ±2.02	19.98 ±2.41	-	2011-2015	Tang et al [39]**
960	Forest	8.98	-	-	2014-2018	State Forestry Administration
204.3	Forest	9.89 ±0.42<br>7.42 ±0.73	20.40 ±0.37<br>17.81 ±0.99	30.29 ±0.79<br>25.24 ±1.72	2013<br>2000-2010	This study
171.3	Cropland	0.55 ±0.02	15.77 ±0.57	-	2011-2015	Tang et al [39]
133.69	Cropland	0.003 ±0.001	3.26 ±0.26	3.26 ±0.26	2013	This study
74.3	Shrubland	0.71 ±0.23	5.91 ±0.43	-	2011-2015	Tang et al [39]
67.63	Shrubland	0.97 ±0.002	4.61 ±0.008	5.57 ±0.008	2013	This study
35.6	Wetland	0.27	6.18	-	2007-2009	Zheng et al [53]
30.73	Wetland	0.28 ±0.001	10.20 ±0.001	10.30 ±0.001	2013	This study
355.05	Grassland	1.61	29.37 ±1.2	30.98 ±1.25	2003-2014	Ma et al [54]
167.0-405.9	Grassland	0.56-4.66	16.7-53.72	17.26-58.38	1980s-1990s	Ma et al [54]***
337.79	Grassland	1.73 ±0.002	39.21 ±0.04	40.93 ±0.04	2013	This study

*area used for C stock calculation;**litter C excluded; **publication reported values collected from references [55–59].

8. Experimental designed for model simulations

We designed three groups of experiments to quantify each major driver's impacts on the terrestrial C stock. Specifically, the first group includes two experiments (Group-1 in Table S10), which were used to examine the historical accumulated impacts of LUCC on the terrestrial C stock over the entire study period from 1900 to 2019. The first experiment (i.e. Allcomb, S1 in Table S10) was designed to let all environmental factors vary from 1900 to 2019, while the second experiment (S2 in Table S10) kept LUCC constant at the 1900 level. The historical impacts of LUCC could be quantified by comparing experiments S1 and S2 (Figure S21). Note that historical C stock change is not equivalent to the sum of factorial attributions as the baseline condition differs. Specifically, the historical C stock change was calculated from the difference of C stock in 2019 and 1980 for the period 1980-2019 (i.e. experiment S1), while in comaprison the total of factorial attribution was calculated from the C stock in 2019 from two experiments (i.e. S1 and S9 in Table S10 and Figure S21).

Moreover, two additional groups of experiments (Group-2&3 in Table S10) were also designed to quantify the effects of each major driver for the recent four decades (1980-2019), during which the forest recovery was initiated by the Chinese government. Specifically, the two groups of experiment simulations were designed to examine the direct and interactive contributions of each major driver (e.g. LUCC, climate, N deposition, rising CO_2, and forest management) to the changes of terrestrial C stock in China since 1980. For example, Group-2 experiments (S3-S8 in Table S10) were designed to keep a specific environmental factor fixed at the 1980 level, while varying other drivers during the entire study period. Conversely, Group-3 experiments (S9-S15 in Table S10) were designed to let at most one environmental factor vary during the period 1980-2019, while keeping the other factors constant at the 1980 level. Note that experiment S10 was designed to keep all environmental factors constant at the 1980 level, which served as a baseline condition for comparison with other experiments in Group-3. The historical impact of LUCC and the contribution of LUCC impacts derived from the factorial experiments are illustrated in Figure S21.

To examine whether the difference of our DLEM simulations and the MIPs (i.e. MsTMIP and TRENDY) was caused by LUCC database or the model used, we set up

DLEM simulations using LUH2 forcing data (other drivers are the same as in Table S10). The LUH2-based LUCC-induced C change was extracted from experiments S1 and S2 but forced by LUH2 database. Additionally, for uncertainty analysis, we designed two more sets of experiments (Table S10) with parameters varying by 1 standard deviation. All the simulations were performed at a 0.5 × 0.5 degree.

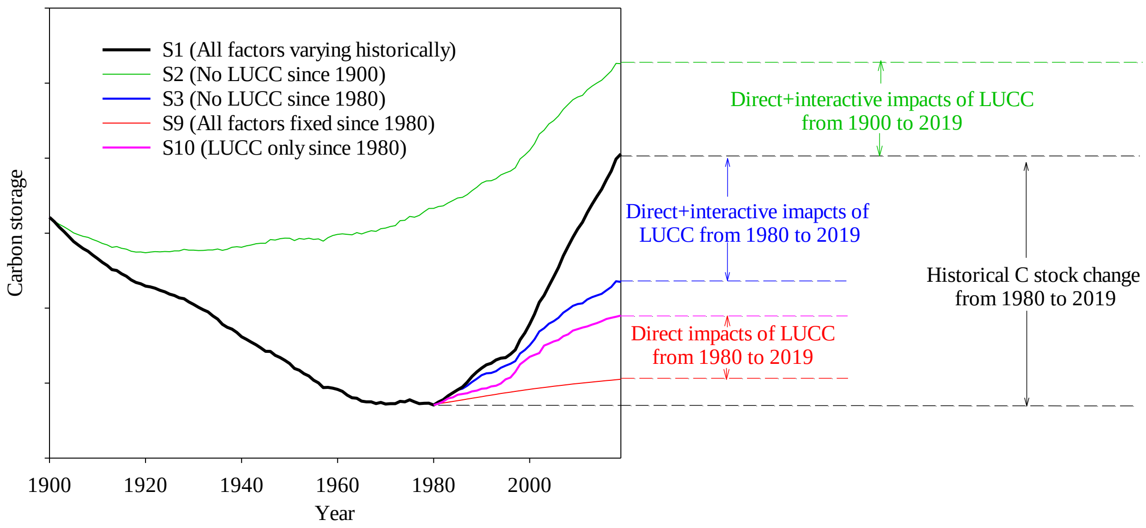

Figure S21. Conceptual diagram to demonstrate the approaches used in quantifying historical C stock change and the impacts of LUCC in a given period in this study. (S1: Allcomb experiment with all environmental factors vary from 1900 to 2019; S2: experiment with LUCC constant at the 1900 level; S3: experiment with LUCC fixed at the 1980 level; S9: experiment with all driving factors fixed constant at the 1900 level; S10: experiment with LUCC varied historically but other factors were fixed constant at the 1980 level since 1980; experiment details can be found in Table S10)

Table S10. Experiments designed for model simulations*

Group	Experiments	Abbr	Drivers					
			LUCC	Climate	Ndep	CO_2	PF management	Nfer
1	Allcomb	S1	1900-2019	1900-2019	1900-2019	1900-2019	1900-2019	1900-2019
	NoLUC	S2	1900	1900-2019	1900-2019	1900-2019	1900-2019	1900-2019
2	NoLUCC	S3	1900-1980	1900-2019	1900-2019	1900-2019	1900-2019	1900-2019
	NoClimate	S4	1900-2019	1900-1980	1900-2019	1900-2019	1900-2019	1900-2019
	NoNdep	S5	1900-2019	1900-2019	1900-1980	1900-2019	1900-2019	1900-2019
	NoCO2	S6	1900-2019	1900-2019	1900-2019	1900-1980	1900-2019	1900-2019
	NoMan	S7	1900-2019	1900-2019	1900-2019	1900-2019	1900-1980	1900-2019
	NoFer	S8	1900-2019	1900-2019	1900-2019	1900-2019	1900-2019	1900-2019
3	NoAll	S9	1900-1980	1900-1980	1900-1980	1900-1980	1900-1980	1900-1980
	LUCC only	S10	1900-2019	1900-1980	1900-1980	1900-1980	1900-1980	1900-1980
	Climate only	S11	1900-1980	1900-2019	1900-1980	1900-1980	1900-1980	1900-1980
	Ndep only	S12	1900-1980	1900-1980	1900-2019	1900-1980	1900-1980	1900-1980
	CO2 only	S13	1900-1980	1900-1980	1900-1980	1900-2019	1900-1980	1900-1980
	PFman only	S14	1900-1980	1900-1980	1900-1980	1900-1980	1900-2019	1900-1980
	Fer only	S15	1900-1980	1900-1980	1900-1980	1900-1980	1900-1980	1900-2019

*experiments S2-S15 are used for attribution analyses of each driver; Group-2 and Group-3 experiments are used for quantification of the direct and interactive impacts; Ndep: nitrogen deposition; Nfer: include nitrogen fertilizer and manure application in cropland.

References

1. Yu, Z., Jin, X., Miao, L. & Yang, X. A historical reconstruction of cropland in China from 1900 to 2016. *Earth Syst. Sci. Data Discuss.* **13**, 3203–3218 (2021).
2. Yu, Z. & Lu, C. Historical cropland expansion and abandonment in the continental U.S. during 1850 to 2016. *Glob. Ecol. Biogeogr.* **27**, 322–333 (2018).
3. Chen, J., Chen, J. & Liao, A. *Remote sensing mapping of global land cover (in Chinese).* (Science Press, 2016).
4. Jun, C., Ban, Y. & Li, S. Open access to Earth land-cover map. *Nature* **514**, 434 (2014).
5. Gong, P., Li, X. & Zhang, W. 40-Year (1978–2017) human settlement changes in China reflected by impervious surfaces from satellite remote sensing. *Sci. Bull.* **64**, 756–763 (2019).
6. Loveland, T. R. *et al.* Development of a global land cover characteristics database and IGBP DISCover from 1 km AVHRR data. *Int. J. Remote Sens.* **21**, 1303–1330 (2000).
7. Hansen, M. C., Sohlberg, R., Defries, R. S. & Townshend, J. R. G. Global land cover classification at 1 km spatial resolution using a classification tree approach. *Int. J. Remote Sens.* **21**, 1331–1364 (2000).
8. Bartholomé, E. & Belward, A. S. GLC2000: A new approach to global land cover mapping from earth observation data. *Int. J. Remote Sens.* **26**, 1959–1977 (2005).
9. Ran, Y. & Li, X. *Comparison report of the four 1-km land cover products of China*. (2006).
10. Ran, Y. Land cover products of China. *Natl. Tibet. Plateau Data Cent.* (2013).
11. Gong, P. *et al.* Stable classification with limited sample: transferring a 30-m resolution sample set collected in 2015 to mapping 10-m resolution global land cover in 2017. *Sci. Bull.* **64**, 370–373 (2019).
12. Yang, X., Jin, X., Guo, B., Long, Y. & Zhou, Y. Research on reconstructing spatial distribution of historical cropland over 300 years in traditional cultivated regions of China. *Glob. Planet. Change* **128**, 90–102 (2015).
13. Hansen, M. C. *et al.* High-resolution global maps of 21st-century forest cover change. *Science* **342**, 850–853 (2013).
14. Li, C. *et al.* A circa 2010 thirty meter resolution forest map for China. *Remote Sens.* **6**, 5325–5343 (2014).
15. Niu, Z. G. *et al.* Mapping wetland changes in China between 1978 and 2008. *Chinese Sci. Bull.* **57**, 2813–2823 (2012).
16. Yu, Z. *et al.* Mapping forest type and age in China's plantations. *Sci. Total Environ.* **744**, 140790 (2020).
17. Liu, S., Wu, S. & Wang, H. Managing planted forests for multiple uses under a changing environment in China. *New Zeal. J. For. Sci.* **44**, S3 (2014).
18. FAO. *Global Forest Resources Assessment 2015*. *FAO Forestry* (2016).
19. Jung, M., Henkel, K., Herold, M. & Churkina, G. Exploiting synergies of global land cover products for carbon cycle modeling. *Remote Sens. Environ.* **101**, 534–553 (2006).
20. Wei, Y. *et al.* The North American carbon program multi-scale synthesis and terrestrial model intercomparison project–Part 2: Environmental driver data. *Geosci. Model Dev.* **6**, 2121–2133 (2014).

21. Hurtt, G. C. *et al.* Harmonization of land-use scenarios for the period 1500-2100: 600 years of global gridded annual land-use transitions, wood harvest, and resulting secondary lands. *Clim. Change* **109**, 117–161 (2011).
22. Winkler, K., Fuchs, R., Rounsevell, M. & Herold, M. Global land use changes are four times greater than previously estimated. *Nat. Commun.* **12**, 2501 (2021).
23. Houghton, R. A. & Nassikas, A. A. Global and regional fluxes of carbon from land use and land cover change 1850–2015. *Global Biogeochem. Cycles* **31**, 456–472 (2017).
24. Liu, M. & Tian, H. China's land cover and land use change from 1700 to 2005: Estimations from high-resolution satellite data and historical archives. *Global Biogeochem. Cycles* **24**, GB3003 (2010).
25. Ramankutty, N. & Foley, J. A. Estimating historical changes in global land cover: Croplands from 1700 to 1992. *Global Biogeochem. Cycles* (1999).
26. Li, H., Lei, Y., Zeng, W. & others. Forest carbon storage in China estimated using forestry inventory data. *Sci. Silvae Sin.* **47**, 7–12 (2011).
27. Guo, Z. *et al.* Spatio-temporal changes in biomass carbon sinks in China's forests from 1977 to 2008. *Sci. China Life Sci.* **43**, 421–431 (2013).
28. Wu, Q. B. *et al.* Carbon sequestration and its potential by forest ecosystem in China. *Acta Ecol. Sin.* **28**, 517–524 (2008).
29. Fang, J., Chen, A., Peng, C., Zhao, S. & Ci, L. Changes in forest biomass carbon storage in China between 1949 and 1998. *Science* **292**, 2320–2322 (2001).
30. Zhang, Y., Wang, X., Pu, Y. & Zhang, J. Changes in forest resource carbon storage in China between 1949 and 2018. *J. Beijing For. Univ.* **43**, 1–14 (2021).
31. National Forestry and Grassland Administration. *Ninth National Forest Resource Inventory Report (2014-2018).* (China Forestry Publishing House, 2019).
32. Wang, X. C., Qi, G., Yu, D. P., Zhou, L. & Dai, L. M. Carbon storage, density, and distribution in forest ecosystems in Jilin Province of Northeast China. *Chinese J. Appl. Ecol.* **22**, 2013–2020 (2011).
33. Liu, S., Xia, C., Feng, W., Zhang, K. & Ma, L. Estimation of vegetation carbon storage and density of forests at tree layer in Tibet, China. *Chinese J. Appl. Ecol.* **28**, 3127–3134 (2017).
34. Li, M., Du, M. & Yu, L. Carbon Storage and Density of Forest Vegetation and Its Spatial Distribution Pattern in Guizhou Province. *J. Northwest For. Univ.* **31**, 48–54 (2016).
35. Ma, Q., Liu, K. & Zhang, H. Carbon storage by forest vegetation and its spatial distribution in Shaanxi. *Resour. Sci.* **34**, 1781–1789 (2012).
36. Zhang, C. H. *et al.* Biomass carbon stocks and dynamics of forests in Heilongjiang Province from 1973 to 2013. *China Environ. Sci.* **38**, 4678–4686 (2018).
37. Spawn, S. A., Sullivan, C. C., Lark, T. J. & Gibbs, H. K. Harmonized global maps of above and belowground biomass carbon density in the year 2010. *Sci. Data* **7**, 112 (2020).
38. Ruesch, A. & Gibbs, H. New IPCC Tier-1 Global Biomass Carbon Map for the Year 2000. (2008).
39. Tang, X. *et al.* Carbon pools in China's terrestrial ecosystems: New estimates based on an intensive field survey. *Proc. Natl. Acad. Sci. U. S. A.* **115**, 4021–4026 (2018).

40. Hengl, T. *et al.* SoilGrids1km - Global soil information based on automated mapping. *PLoS One* **9**, e105992 (2014).
41. Liu, F. *et al.* Mapping high resolution National Soil Information Grids of China. *Sci. Bull.* **67**, 328–340 (2021).
42. Zhao, M., Yue, T., Zhao, N., Sun, X. & Zhang, X. Combining LPJ-GUESS and HASM to simulate the spatial distribution of forest vegetation carbon stock in China. *J. Geogr. Sci.* **24**, 249–268 (2014).
43. Xu, L. *et al.* Carbon storage in China's terrestrial ecosystems: A synthesis. *Sci. Rep.* **8**, 2806 (2018).
44. Xu, X. L., Cao, M. K. & Li, K. R. Temporal-spatial dynamics of carbon storage of forest vegetation in China. *Prog. Geogr.* **26**, 1–10 (2007).
45. Zhang, C. *et al.* China's forest biomass carbon sink based on seven inventories from 1973 to 2008. *Clim. Change* **118**, 933–948 (2013).
46. Xu, B., Guo, Z. Di, Piao, S. L. & Fang, J. Y. Biomass carbon stocks in China's forests between 2000 and 2050: A prediction based on forest biomass-age relationships. *Sci. China Life Sci.* **53**, 776–783 (2010).
47. Houghton, R. A. Temporal patterns of land-use change and carbon storage in China and tropical Asia. *Sci. China Ser. C Life Sci.* **45**, 10–17 (2002).
48. Houghton, R. A. & Hackler, J. L. Sources and sinks of carbon from land-use change in China. *Global Biogeochem. Cycles* **17**, 1034 (2003).
49. Tian, H. *et al.* China's terrestrial carbon balance: Contributions from multiple global change factors. *Global Biogeochem. Cycles* **25**, GB1007 (2011).
50. Lai, L. *et al.* Carbon emissions from land-use change and management in China between 1990 and 2010. *Sci. Adv.* **2**, e1601063 (2016).
51. Liu, J., Wang, S., Chen, J. M., Liu, M. & Zhuang, D. Storages of soil organic carbon and nitrogen and land use changes in China: 1990-2000. *Acta Geogr. Sin.* **59**, 483–496 (2004).
52. Shangguan, W. *et al.* A China data set of soil properties for land surface modeling. *J. Adv. Model. Earth Syst.* **5**, 212–224 (2013).
53. Zheng, Y., Niu, Z., Gong, P., Dai, Y. & Shangguan, W. Preliminary estimation of the organic carbon pool in China's wetlands. *Chinese Sci. Bull.* **58**, 662–670 (2013).
54. Ma, A., He, N., Yu, G., Wen, D. & Peng, S. Carbon storage in Chinese grassland ecosystems: Influence of different integrative methods. *Sci. Rep.* **6**, 21378 (2016).
55. Ni, J. Carbon storage in terrestrial ecosystems of China: Estimates at different spatial resolutions and their responses to climate change. *Clim. Change* **49**, 339–358 (2001).
56. Ni, J. Carbon storage in grasslands of China. *J. Arid Environ.* **50**, 205–218 (2002).
57. Fang, J. Y., Guo, Z. Di, Piao, S. L. & Chen, A. P. Terrestrial vegetation carbon sinks in China, 1981-2000. *Sci. China, Ser. D Earth Sci.* **50**, 1341–1350 (2007).
58. Yang, Y., Fang, J., Ma, W., Guo, D. & Mohammat, A. Large-scale pattern of biomass partitioning across China's grasslands. *Glob. Ecol. Biogeogr.* **19**, 268–277 (2010).
59. Piao, S., Fang, J., Zhou, L., Tan, K. & Tao, S. Changes in biomass carbon stocks in China's grasslands between 1982 and 1999. *Global Biogeochem. Cycles* **21**, GB2002 (2007).
