# Supplementary material for: Forest expansion dominates China’s land carbon sink since 1980
Source: Nat Commun. 2022 Sep 13;13:5374. doi: 10.1038/s41467-022-32961-2 (PMC9470586; doi:10.1038/s41467-022-32961-2)
Supplement: Supplementary file 2 — Description of Additional Supplementary Files [file 41467_2022_32961_MOESM2_ESM.pdf]

Description of Additional Supplementary Files

File Name: Supplementary Data 1
Description: The reconstructed LUCC data used in this study.
